# Predicting Pharmacokinetics of Drugs in Patients with Heart Failure and Optimizing Their Dosing Strategies Using a Physiologically Based Pharmacokinetic Model

**DOI:** 10.3390/pharmaceutics17111394

**Published:** 2025-10-28

**Authors:** Weiye Gu, Qingxuan Shao, Ling Jiang

**Affiliations:** Center of Drug Metabolism and Pharmacokinetics, School of Pharmacy, China Pharmaceutical University, Nanjing 210009, China; 3322010288@stu.cpu.edu.cn (W.G.); 3323010653@stu.cpu.edu.cn (Q.S.)

**Keywords:** physiologically based pharmacokinetic model, heart failure, pharmacokinetics, global sensitivity analysis, dose optimization

## Abstract

**Background:** Heart failure (HF), as the end stage of various cardiac diseases, alters blood flow to key organs responsible for drug clearance. This can lead to unpredictable and often suboptimal drug exposure, creating a critical need for quantitative tools to guide precise dosing in this vulnerable population. **Methods**: This study aimed to establish a whole-body physiologically based pharmacokinetic (PBPK) model for characterizing drug pharmacokinetics in both healthy subjects and patients across the HF severity spectrum. Eight commonly used drugs (digoxin, furosemide, bumetanide, torasemide, captopril, valsartan, felodipine and midazolam) for treating HF and its comorbidities were selected. Following successful validation against clinical data from healthy subjects, the PBPK model was extrapolated to HF patients. Pharmacokinetics of the eight drugs in 1000 virtual HF patients were simulated by replacing tissue blood flows and compared using clinical observations. **Results**: Most of the observed concentrations were encompassed within the 5th–95th percentiles of simulated values from 1000 virtual HF patients. Predicted area under the concentration–time curve and maximum plasma concentration fell within the 0.5~2.0-fold range relative to clinical observations. Sensitivity analysis demonstrated that intrinsic renal clearance, unbound fraction in blood, muscular blood flow, and effective permeability coefficient significantly impact plasma exposure of digoxin at a steady state. Oral digoxin dosing regimens for HF patients were optimized via the validated PBPK model to ensure that steady-state plasma concentrations in all HF patients remain below the toxicity threshold (2.0 ng/mL). **Conclusions:** A PBPK model was successfully developed to predict the plasma concentration–time profiles of the eight tested drugs in both healthy subjects and HF patients. Furthermore, this model may also be applied to guide digoxin dose optimization for HF patients.

## 1. Introduction

Heart failure (HF) represents the terminal pathological convergence of diverse cardiac and extracardiac disorders, manifesting as a multifaceted clinical syndrome exhibiting phenotypic heterogeneity. HF is classified into functional classes I–IV according to the New York Heart Association (NYHA)’s criteria [1,2]. Patients with HF commonly exhibit concomitant systemic hypertension and/or coronary artery disease [3], leading to hemodynamic redistribution across hepatic, renal, and gastrointestinal circulations. Hemodynamic quantification studies revealed progressive perfusion deficits in NYHA class II–IV patients, with hepatic perfusion at 76%, 54%, and 46% of age-matched controls, and renal perfusion at 78%, 55%, and 63%, respectively [2]. HF demonstrates significant comorbidity clustering, with diabetes mellitus, chronic obstructive pulmonary diseases, and anemia representing the predominant concurrent conditions. HF-induced hemodynamic perturbations and multiorgan dysfunction affecting hepatic, renal, and gastrointestinal systems critically modulate the pharmacokinetic–pharmacodynamic profiles of therapeutic agents and comorbidity treatments. The effects of HF on drug pharmacokinetics exhibit substantial variability. For instance, patients with HF demonstrate a 29% reduction in D-xylose absorption (reflecting carrier-mediated transport activity) alongside a 35% increase in intestinal permeability (assessed via urinary 5 h lactulose/mannitol ratio) compared to healthy subjects [4]. Similarly, the pharmacokinetics of midazolam are altered in HF, exhibiting a 30% decrease in systemic clearance and a prolonged elimination half-life [5]. While oral absorption of torasemide remains unchanged in HF patients, time to peak concentration increases from 0.9 h in healthy subjects to 1.7 h in HF patients. The oral clearance, renal, and non-renal clearances of torasemide are, respectively, reduced by 44%, 38%, and 46% of healthy subjects, leading to increases in plasma exposure of torasemide by 107% [6]. These collective factors confer significant complexity to pharmacotherapeutic optimization in HF patients, thereby creating substantial challenges for drug dosing and efficacy attainment. The physiologically based pharmacokinetic (PBPK) modeling integrates human physiological parameters with drug-specific physicochemical properties to predict drug pharmacokinetics in patients, serving as an advanced methodology for clinical pharmacokinetic prediction in this population [7,8].

The objective of this study was to develop a PBPK model that accounts for HF-induced alterations in tissue perfusion and hepatic/renal functions to predict drug pharmacokinetics in HF patients. Eight therapeutic agents were selected for evaluation: a cardiac glycoside (digoxin); diuretics (furosemide, bumetanide, and torasemide); an angiotensin-converting enzyme inhibitor (captopril); an angiotensin II receptor antagonist (valsartan); a calcium channel blocker (felodipine); and a sedative-hypnotic (midazolam). This selection was designed to cover a wide range of elimination pathways (renal and hepatic) and physicochemical properties, providing a robust test for the model’s capability. Of particular interest was digoxin, a narrow-therapeutic-index drug whose complex pharmacokinetics have been the subject of numerous PBPK studies, making it a critical model drug to test the impact of HF-specific hemodynamic changes. The model predictions were validated against clinical pharmacokinetic data obtained from HF patients. A virtual clinical trial was subsequently conducted using the validated PBPK model to enable dose optimization in HF patients through comparative assessment of plasma exposure profiles between HF patients and healthy subjects.

## 2. Methods

### 2.1. General Workflow

**Figure 1:**
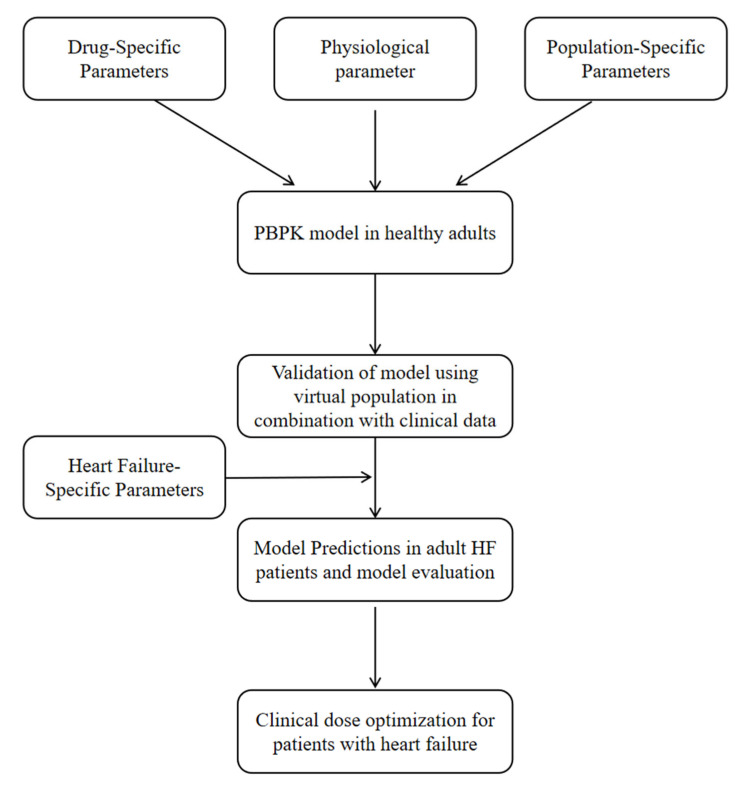
Workflow for PBPK model development and application. A PBPK model was developed and validated using clinical pharmacokinetic data from healthy subjects. The model was then adapted to HF patients by incorporating disease-specific physiological parameters, and was validated against pharmacokinetic data from HF patients. Finally, the validated model was employed to optimize dosing regimens in HF patients.

The PBPK modeling workflow for HF patients is schematically presented in Figure 1. First, a whole-body PBPK model was constructed to simulate drug pharmacokinetics in a virtual healthy population, with validation performed using clinical pharmacokinetic data. Subsequently, the model was adapted to HF patients by modifying system-specific physiological parameters. Pharmacokinetic predictions were conducted in 1000 virtual HF patients and compared with clinical pharmacokinetic data from the literature. Ultimately, the validated model was subsequently applied to explore dose optimization strategies in HF patients.

### 2.2. Model Development

**Figure 2:**
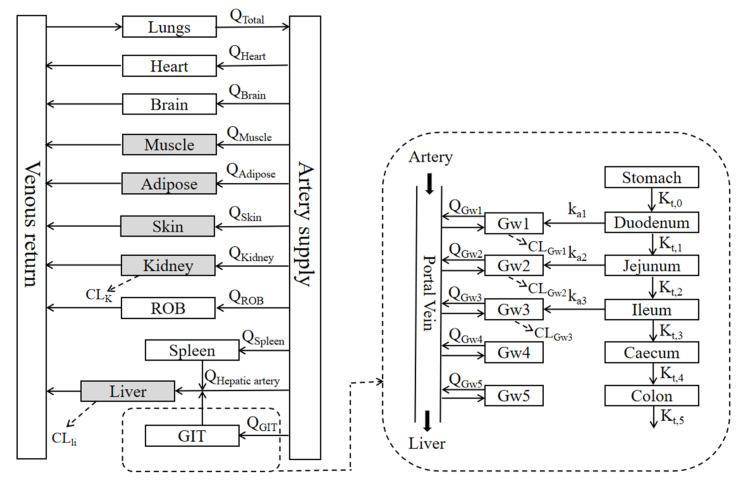
Structure of the PBPK model. Q, blood flow; k_ai_, constant of absorption rate; K_t,0_, constant of gastric emptying rate; K_t,i_, constant of intestinal transit rate; CL_li_, hepatic clearance; CL_K_, renal clearance; CL_Gwi_, intestinal clearance (duodenum, jejunum and ileum). The gray-shaded areas indicate the tissues and organs that demonstrate blood-flow alterations in HF patients.

A PBPK model was developed to concurrently predict the pharmacokinetics of eight commonly used drugs in HF patients (Figure 2). According to the anatomical structure of body, the PBPK model consists of the stomach, intestine, liver, kidney, lung, heart, brain, muscle, adipose, skin, arterial blood, venous blood, and the rest of body (ROB), which are connected by the blood circulation system. The intestine is anatomically divided into the duodenum, jejunum, ileum, caecum, and colon, each comprising an intestinal wall and lumen. It was assumed that drug absorption would only occur at the duodenum, jejunum, and ileum, and that intestinal absorption of the drug would be a first-order process. The corresponding mass equations are illustrated in the Supplementary Information.

### 2.3. Model Development in HF Patients

The coding and solving of the PBPK model were conducted using Phoenix WinNonlin software (Version 8.4, Certara, Radnor, PA, USA). All of the available information on anatomical and physiological parameters in 70 kg healthy subjects and the pharmacokinetic parameters of the tested drugs were collected for the initial model construction (Table S1). K_t:p_ values of the indicated drugs were estimated using Rodgers’ and Ruark’s methods [9,10]. Pharmacokinetic parameters of the indicated drugs were directly cited from the literature/website or were estimated/optimized from predicted pharmacokinetic data using Phoenix WinNonlin 8.4.

The PBPK model was first validated using clinical observations from healthy subjects. Subsequently, it was adapted for HF patients by replacing the anatomical and physiological parameters of healthy individuals with those specific to HF patients. It was assumed that HF mainly affected total blood flow and tissue blood flow without affecting the intrinsic clearance of drugs in the liver and kidneys. The cardiac output and tissue blood flow in HF patients were estimated using the relationship between central hemodynamics and regional blood flow in normal subjects and in HF patients according to previous reports [2,11,12], i.e., hepatic blood flow in patients with mild, moderate, and severe heart failure declined to 76%, 54%, and 46% of baseline levels, respectively; renal blood flow was reduced to 78%, 55%, and 63% of baseline levels; while blood flow to the skin, adipose tissue, and muscle decreased to 57%, 44%, and 28% of baseline levels [2]. Alterations in the gastrointestinal and splenic blood flow were assumed to be in parallel with alterations in hepatic blood flow. The calculated tissue blood flows in HF patients are listed in Table 1.

The simulations incorporated four virtual populations (normal population and HF patients with grade II, grade III, and grade IV), each comprising 1000 independently generated virtual individuals (weighing 70 kg). It was assumed that the physiologic parameters (such as tissue volumes and blood flows) were held constant across all subjects; each virtual subject was created through systematic sampling of five key parameters: CL_li,int_, CL_k,int_, f_u,b_, P_eff_, and k_a_. These parameters were randomly sampled within 80–120% of their baseline values to establish individual variability. The exponential model and multiplicative residual error model were used to simulate the inter-individual and intra-individual variability of these parameters. The first-order conditional estimation-extended least squares algorithm was implemented as the computational engine for parameter estimation. Pharmacokinetic characterization was performed using 10 independent Monte Carlo simulations in a virtual cohort of 1000 subjects, ensuring reliable population variability analysis. This iterative process enabled the derivation of reliable pharmacokinetic reference ranges, specifically the 5th, 50th, and 95th percentiles, using population-level aggregation of simulation outcomes.

### 2.4. Criterion of the Developed PBPK Model

The PBPK model was considered to be successful (1) if the simulated area under the curve (AUC) or maximum plasma concentration (C_max_) fell within 0.5~2.0-fold range of the clinical observations or (2) the observations were within the 5th–95th percentiles of the simulation derived from 1000 virtual subjects.

Effects of HF on the plasma exposure of the tested drugs were indexed as the ratio of AUC in HF patients to that in healthy subjects (AUCR) or the ratio of C_max_ in HF patients to that in healthy subjects (C_max_R), i.e.,

(1) $$AUCR=\frac{{AUC}_{HF}}{{AUC}_{H}}$$

(2) $$AUCR=\frac{{CL}_{H}}{{CL}_{HF}}$$

(3) $$AUCR=\frac{{CL}_{H}}{{CL}_{HF}}$$

where AUC_HF_, AUC_H_, CL_HF_, CL_H_, C_max,HF_, and C_max,H_ represent the AUC, CL, and C_max_ of the tested drugs in HF patients and healthy subjects, respectively.

## 3. Results

### 3.1. Drug Data Set

The tested drugs were collected from publications on PubMed based on the following criteria: (1) The drugs were orally or intravenously administered to both healthy subjects and HF patients. (2) Formulations for oral administration of the indicated drug were immediately released. (3) Plasma concentration–time profiles or their main plasma exposure parameters such as AUC or C_max_ were shown. (4) The pharmacokinetics of the indicated drugs in healthy subjects and HF patients could be derived from different clinic reports. Eight drugs, including digoxin, furosemide, bumetanide, torasemide, captopril, valsartan, felodipine, and midazolam, were selected to validate the developed PBPK model for HF patients. The description of the tested drugs is shown in the Supplementary Materials. The collected pharmacokinetic parameters of the drugs and the drugs’ information in clinical reports are listed in Tables S2 and S3.

### 3.2. Development of PBPK Model and Validation in Healthy Subjects

**Figure 3:**
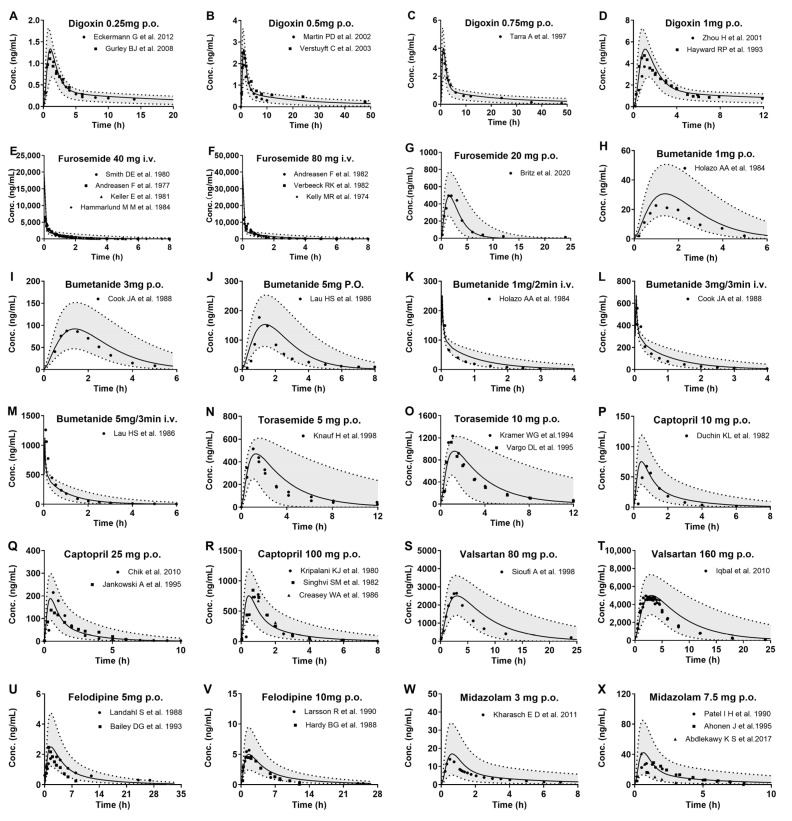
The observed (points) and predicted (lines) plasma concentrations of the tested drugs following intravenous or oral administration to healthy subjects. Digoxin following oral 0.25 mg (**A**) [13,14], 0.5 mg (**B**) [15,16], 0.75 mg (**C**) [17], and 1 mg (**D**) [18,19]; furosemide following intravenous 40 mg (**E**) [20,21,22,23], 80 mg (**F**) [24,25,26], and oral 20 mg (**G**) [27]; bumetanide following oral 1 mg (**H**) [28], 3 mg (**I**) [29], and 5 mg (**J**) [30], and intravenous 1 mg (**K**) [28], 3 mg/3 min (**L**) [29], and 5 mg/3 min (**M**) [30]; torasemide following oral 5 mg (**N**) [31] and 10 mg (**O**) [6,32]; captopril following oral 10 mg (**P**) [33], 25 mg (**Q**) [34,35], and 100 mg (**R**) [36,37,38]; valsartan following oral 80 mg (**S**) [39] and 160 mg (**T**) [40]; felodipine following oral 5 mg (**U**) [41,42] and 10 mg (**V**) [43,44]; midazolam following oral 3 mg (**W**) [45] and 7.5 mg (**X**) [5,46,47]. Solid line: 50th percentile of simulated plasma concentrations; Shadow: 5th–95th interval of the simulated plasma concentrations.

Plasma concentration–time profiles of the eight tested drugs following intravenous injection or oral administration to healthy subjects were simulated using the developed PBPK model and compared with clinical observations (Figure 3). The information of drugs and healthy subjects is shown in Table S3. The results show that most of the observed data of the tested agents fell within the 5th–95th percentiles of the simulated data. Plasma concentrations were estimated using the simulated data derived from 1000 virtual individuals and compared with clinical observations. Except for overestimation in AUC of midazolam, almost 70/71 of simulated pharmacokinetic parameters (AUC and C_max_) values for the eight tested drugs were within 0.5~2.0-fold range relative to observations (Table S4 and Figure 5). All of the results demonstrate that the PBPK model was successfully developed.

### 3.3. Development of PBPK Model and Validation in HF Patients

**Figure 4:**
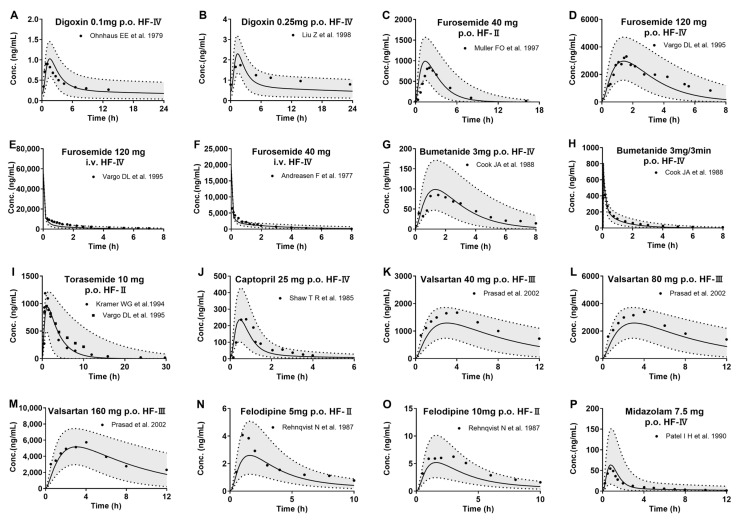
The observed (points) and predicted (lines) plasma concentrations of the tested drugs following intravenous or oral administration to HF patients. Digoxin following oral 0.1 mg (**A**) [48], 0.25 mg (**B**) [49]; furosemide following oral 40 mg (**C**) [50] and 120 mg (**D**) [32], and intravenous 120 mg (**E**) [32] and 40 mg (**F**) [21]; bumetanide following oral 3 mg (**G**) [29] and intravenous 3 mg/3 min (**H**) [29]; torasemide following oral 10 mg (**I**) [6,32]; captopril following oral 25 mg (**J**) [51]; valsartan following oral 40 mg (**K**) [52], 80 mg (**L**) [52], and 160 mg (**M**) [52]; felodipine following oral 5 mg and (**N**) [53] 10 mg (**O**) [53]; midazolam following oral 7.5 mg (**P**) [5]. Solid line: 50th percentile of simulated plasma concentrations; Shadow: 5th–95th interval of the simulated plasma concentrations.

**Figure 5:**
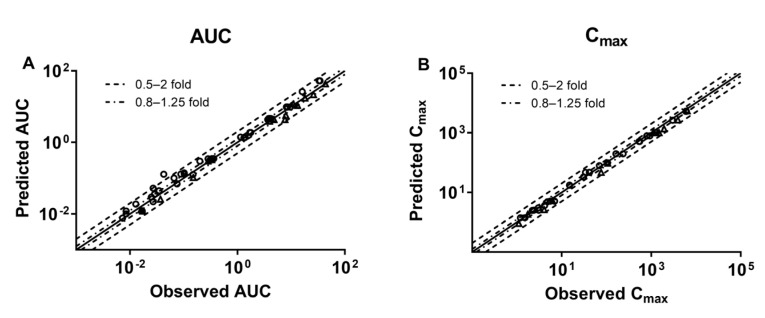
Comparison of the predicted AUC (**A**) and C_max_ (**B**) with observations in healthy subjects (hollow circle) and HF patients (hollow triangle). Solid and dotted lines, respectively, represent unity; the predicted values are within 0.5~2.0-fold range of the observed data, and most are within the more stringent 0.8~1.25-fold range.

The developed PBPK model, following validation in healthy subjects, was used to predict the plasma concentrations of the eight tested drugs following intravenous or oral administration to 1000 virtual HF patients (Figure 4). According to corresponding clinical scenarios, plasma concentration profiles of drugs were simulated and pharmacokinetic parameters (AUC and C_max_) were estimated. The results show that the observed drug concentrations of the eight tested drugs in HF patients were within the 5th–95th percentiles of pharmacokinetic profiles derived from 1000 virtual HF patients. All of the estimated AUC and C_max_ values were also within 0.5~2.0-fold of clinical observations, and most were within the 0.8–1.25-fold range (Figure 5 and Table 2).

**Figure 6:**
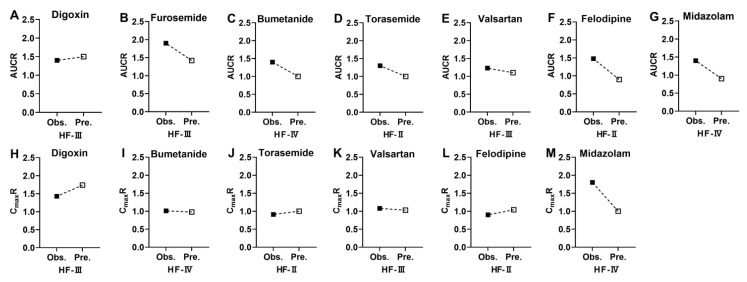
The predicted AUCR/observed AUCR (**A**–**F**) and the predicted C_max_R/observed C_max_R (**G**–**K**) for the tested 8 drugs in HF patients. AUCR of (**A**) Digoxin; (**B**) furosemide; (**C**) bumetanide; (**D**) torasemide; (**E**) valsartan; (**F**) felodipine; (**G**) midazolam. C_max_R of (**H**) Digoxin; (**I**) bumetanide; (**J**) torasemide; (**K**) valsartan; (**L**) felodipine; (**M**) midazolam. The solid and hollow squares correspond to the observed and predicted values, respectively.

Extents of alterations in pharmacokinetic parameters under HF, AUCR, and C_max_R were also predicted using the estimated pharmacokinetic parameters (Figure 6) and compared with clinical observations. AUC or C_max_ values originated from different clinical reports or different doses; thus, the AUC or C_max_ values were normalized by dose. The results show that the vast majority of the predicted AUCR and C_max_R were close to clinical observations. These results collectively demonstrate that the pharmacokinetic profiles of the eight tested drugs in HF patients were successfully predicted using the developed PBPK model.

### 3.4. Sensitivity Analysis of Model Parameters

**Figure 7:**
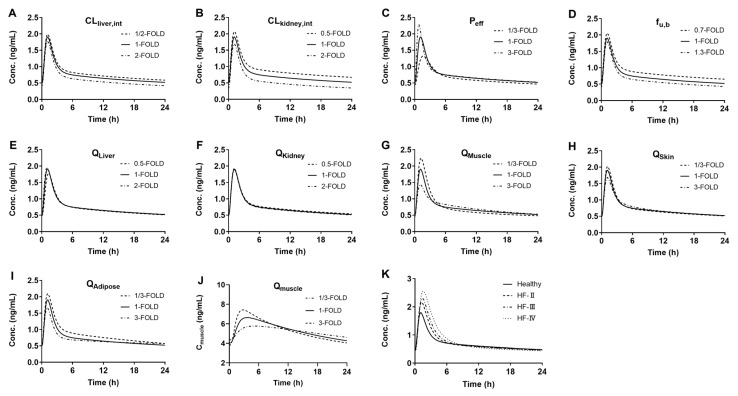
Local sensitivity analysis (**A**–**I**) for corresponding parameters on pharmacokinetic profiles of digoxin at steady-state following oral digoxin (0.25 mg). CL_liver,int_ (**A**), CL_kidney,int_ (**B**), P_eff_ (**C**), f_u,b_ (**D**), Q_L_ (**E**), Q_K_ (**F**), Q_M_ (**G**), Q_S_ (**H**), Q_A_ (**I**), intramuscular drug concentration (**J**), HF severity (**K**).

The plasma concentration–time curve of digoxin at steady-state following oral administration of a multidose of digoxin (0.25 mg) was used as an example for pharmacokinetic sensitivity. Local sensitivity analysis was conducted among the parameters potentially influencing drug pharmacokinetics, including CL_liver,int_, CL_kidney,int_, P_eff_, f_u,b_, Q_L_, Q_K_, Q_S_, Q_M_ and Q_A_. Digoxin shows high affinities to skin, muscle, and adipose tissue. A previous study showed that alterations in muscular blood flow remarkably affect disposition [54]. Thus QS, QM, and QA were also selected for sensitivity analysis. Based on the corresponding parameter changes listed in Table 2, the values of Q_S_, Q_M_, and Q_A_ were varied by one-/three-fold and three-fold, and the variations of Q_L_ and Q_K_ were varied by one-/two-fold and two-fold. According to the parameters reported in the literature (CL_L_ and CL_K_), the calculated variations of CL_liver,int_ and CL_kidney,int_ were mostly within a two-fold range. Therefore, the variation ranges of CL_liver,int_ and CL_kidney,int_ were set to one-/two-fold and two-fold of their original values [55,56]. The reported values of P_eff_ for digoxin in the literature varied widely. Therefore, the P_eff_ value was set to one-/three-fold and three-fold of its original value [55]. The reported f_u,p_ values for digoxin in the literature included 0.61, 0.71, and 0.95, so the variation range of f_u,b_ was set between 0.7-fold and 1.3-fold [9,55,57]. The results show that these measured parameters affected the pharmacokinetic characteristics of the drug to varying degrees (Figure 7). Their contributions to the AUC of digoxin were CL_kidney,int_ > f_u,b_ > CL_liver,int_ > Q_A_ > P_eff_ > Q_M_ > Q_S_ > Q_L_ > Q_K_. The sensitivity analysis identified that Q_M_, which is a parameter notably altered in HF, was among the most sensitive parameters governing systemic exposure. Its contribution to the C_max_ of digoxin was significant. To elucidate the mechanism underlying this sensitivity, the digoxin concentrations in muscle tissue were further simulated (Figure 7J). The results show that the increase in Q_M_ elevated peak digoxin concentration in muscle (C_muscle_), inferring the increases in muscle distribution. On the contrary, reductions in Q_M_ decreased peak digoxin concentration in muscle (C_muscle_), inferring the decreases in muscle distribution. These findings may explain why systemic C_max_ is negatively related to Q_M_. The effects of HF severity (II, III, and IV) on pharmacokinetic profiles of digoxin at steady-state following multidose of digoxin (0.25 mg) were also analyzed. It was consistent with our expectation that plasma exposure to digoxin increased along with HF severity (Figure 7K).

For further systematic evaluation of the sensitivity of various parameters in the PBPK model on output results, we employed the Sobol global sensitivity analysis (GSA) method to quantitatively calculate first-order sensitivity indices and total order sensitivity indices. This approach utilized variance decomposition techniques, not only enabling the identification of individual parameter effects, but also the revelation of comprehensive contributions from nonlinear interactions between parameters to model outputs. Compared to local sensitivity analysis, this method proved more suitable for parameter prioritization in complex models. The technical implementation was developed within the R language ecosystem (version 4.4.3), utilizing RStudio (version 2024.12.1+563) as the integrated development environment for code development and visualization analysis, with compilation environment configuration completed using RTools 4.4. An efficient sampling strategy was adopted to generate multidimensional parameter combination sample sets. These sampled parameters were subsequently incorporated into the PBPK model for batch simulations, during which core pharmacokinetic metrics (AUC) under different parameter combinations were systematically recorded. Based on the simulation data set, variance decomposition techniques were applied to quantify both types of sensitivity indices. Visualization tools were employed to present parameter sensitivity rankings, and bivariate bar charts were generated to comparatively analyze the independent and synergistic influences of each parameter.

**Figure 8:**
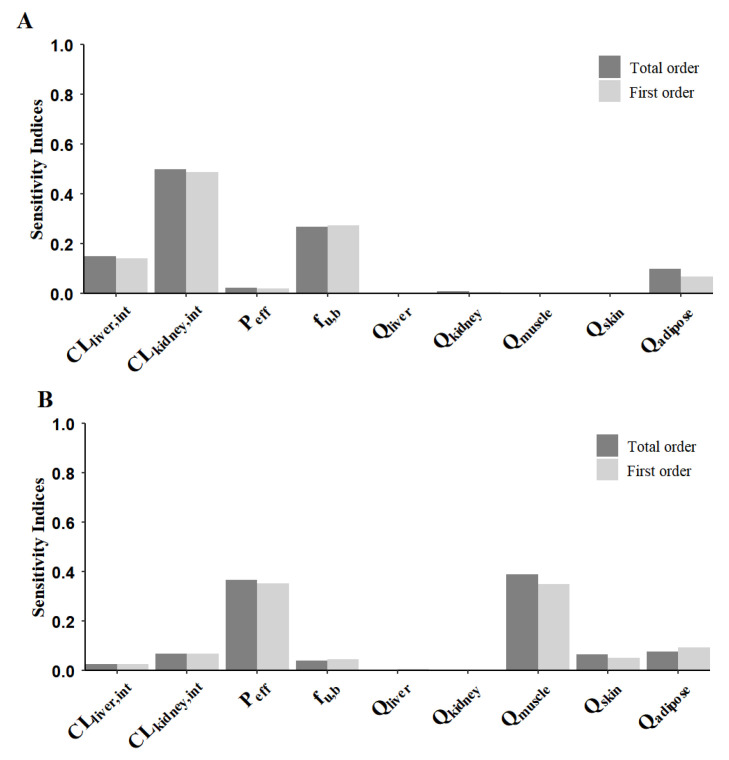
Sobol global sensitivity analysis on AUC (**A**) and C_max_ (**B**).

GSA based on the Sobol method demonstrated significant variations in model output sensitivity to specific physiological and pharmacokinetic parameters. As shown in Figure 8, using AUC (A) as a pharmacokinetic parameter, the SI and ST analysis results reveal that CL_kidney,int_, f_u,b_, CL_liver,int_, and Q_A_ exerted the most substantial influence on model outputs. CL_kidney,int_ exhibited the highest sensitivity in both SI and ST indices, indicating its dominant role in both independent effects and parameter interaction contributions. Meanwhile P_eff_, Q_M_, f_u,b_, Q_L_, and Q_K_ displayed relatively low sensitivity. When C_max_ (B) was used as the pharmacokinetic parameter, Q_M_ and P_eff_ predominated, while the contributions of other parameters were comparable.

### 3.5. Virtual Simulation for Dose Optimization

**Figure 9:**
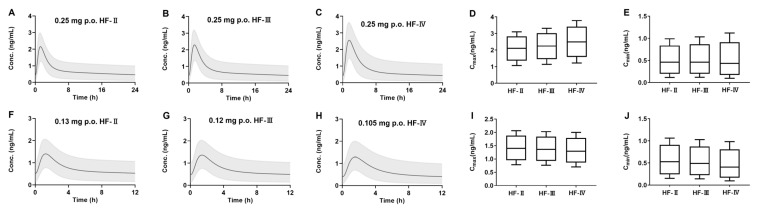
The simulated steady-state plasma concentrations of digoxin following multidose of digoxin to HF II, HF III, and HF IV patients using the developed PBPK model. Plasma concentrations of digoxin following the recommended dosing regimens (qd, 0.25 mg) to (**A**) HF II, (**B**) HF III, and (**C**) HF IV patients; Box-Whisker analysis of digoxin steady-state (**D**) C_max_ and (**E**) C_min_. Plasma concentrations of digoxin according to the adjusted dosage regimens in (**F**) HF II (0.13 mg, Bid), (**G**) HF III (0.12 mg, Bid), and (**H**) HF IV patients (0.105 mg, Bid); Box–Whisker analysis of digoxin steady-state (**I**) C_max_ and (**J**) C_min_. Shadow: 5th–95th interval of the simulated plasma concentrations.

The developed PBPK model was further applied to quantify the impact of HF on drug exposure and optimize the individualized dosing regimens. Digoxin was exemplified. It is generally accepted that the plasma toxic threshold level of digoxin is (2.0 ng/mL) [58,59]. The steady-state plasma concentration of digoxin following the recommended dose of digoxin (0.25 mg, qd) was simulated using the developed PBPK model (Figure 9A–C) and Box-Whisker analysis was also documented (Figure 9D,E). The results show that the steady-state C_max_ of digoxin increased with HF severity. Further study showed that, according to the recommended dosing regimens (qd, 0.25 mg), the steady-state C_max_ of digoxin in over 50% of HF II, III, and IV patients exceeded its toxic threshold (2.0 ng/mL), especially in HF IV patients. The time that concentration of digoxin in HF II, III, and IV patients above 2.0 ng/mL was 0.7 h, 1 h, and 1.7 h, respectively. Clinical practice guidelines for digoxin suggest that serum digoxin levels 0.5–0.9 ng/mL are therapeutic for HF [60]. Moreover, only 43.8% of HF II patients, 44% of HF III patients, and 40.6% of HF IV patients showed concentrations (C_min_) of digoxin over 0.5 ng/mL. These results demonstrate the necessity of dose optimization. To ensure the steady-state C_max_ of digoxin in HF patients below the toxicity threshold (2.0 ng/mL) and the steady-state C_min_ over the minimum effective concentration (0.5 ng/mL), daily oral doses of digoxin were adjusted to 0.13 mg (bid) for HF II patients, 0.12 mg (bid) for HF III patients, and 0.105 mg (bid) for HF IV patients, respectively. Plasma concentrations of digoxin at steady-state following the adjusted dose regimen were simulated (Figure 9F–H), and Box-Whisker analysis was also documented (Figure 9I,J). Data from Box-Whisker analysis showed that the simulated steady-state C_max_ values in all of HF II, III, and IV patients were below 2.0 ng/mL (Figure 9I) and that the simulated steady-state C_min_ values in 51.7% of HF II patients, 47.2% of HF III patients, and 36.7% of HF IV patients were over 0.5 ng/mL (Figure 9J) following the adjusted dosing regimen.

## 4. Discussion

HF, the terminal phase of cardiac disease progression, is characterized by systemic physiological alterations, including reduced cardiac output and compromised tissue perfusion. These pathophysiological changes significantly impact drug pharmacokinetics and clinical efficacy, creating substantial challenges in therapeutic decision-making to balance efficacy and adverse effects. The PBPK model integrates human physiological parameters and drug-specific physicochemical properties, enabling the prediction of drug disposition in HF patients. This approach facilitates optimized dosing strategies to minimize toxicity while maintaining therapeutic effectiveness.

A whole-body PBPK model was developed to simulate drug pharmacokinetics in healthy subjects and HF patients stratified by disease severity. Digoxin, furosemide, bumetanide, torasemide, captopril, valsartan, felodipine, and midazolam served as model drugs. Simulation results demonstrate that most clinical pharmacokinetic observations in healthy and HF patients fell within the 5th–95th percentile ranges derived from 1000 virtual subject simulations. Additionally, most of the predicted AUC and C_max_ values exhibited 0.5~2.0-fold deviations from clinical data. These findings demonstrate the successful development of the PBPK model for predicting the pharmacokinetics of the investigated drugs across both healthy and HF populations.

Digoxin, a narrow therapeutic index drug, served as the subject for sensitivity analysis. Sensitivity analysis revealed that intrinsic renal clearance was the dominant parameter governing variability in steady-state digoxin exposure, followed by the unbound fraction in blood. Notably, although renal elimination and biliary elimination are the primary routes for digoxin clearance, alterations in renal or hepatic blood flow slightly affected plasma exposure of digoxin, which is in line with the fact that digoxin has a low renal extraction and a low hepatic extraction. Alterations in muscle blood flow remarkably affect the plasma exposure of digoxin, which may be attributed to digoxin’s high binding affinity for muscle tissue and the extensive volume distribution of muscle.

An important problem for patients with HF is that some drugs (such as digoxin) for the treatment of HF have a relatively low therapeutic index and narrow safety range. Clinical practice guidelines for digoxin suggest that serum digoxin levels of 0.5–0.9 ng/mL are therapeutic for HF, and that higher levels might be harmful [60]. When the serum digoxin level in patients was ≥2.0 ng/mL, the patients often showed high rates of emergency department visits [58]. The mortality rate of HF patients treated with digoxin is related to the concentration of digoxin. The mortality rates of patients with digoxin levels of less than 1.0 ng/mL, 1.1–2.0 ng/mL, 2.1–4.0 ng/mL, and 4.1–6.0 ng/mL, and more than 6.0 ng/mL were reported to be, respectively, 2.0%, 5.0%, 8.6%, 7.9%, and 50% for a four-week period [59]. Simulations show that, according to the clinically recommended regimen of digoxin (0.25 mg, qd), the steady-state C_max_ of digoxin in over 50% of HF patients exceeds toxic levels (2 ng/mL) and the steady-state C_min_ of digoxin in over 55% of HF patients was below 0.5 ng/mL, demonstrating the necessity of dose regimen adjustment in HF patients. To ensure both a steady-state C_max_ below 2 ng/mL and steady-state C_min_ over 0.5 ng/mL, the dosage regimens of digoxin for Class II, III, and IV HF patients were optimized using the validated PBPK model. The simulation that it was impossible to ensure both steady-state C_max_ below 2 ng/mL and steady-state C_min_ over 0.5 ng/mL based on the dose regimen once a day; thus, medication frequency was set to be twice a day. The results show that, according to the adjusted dose regimen of digoxin, steady-state C_max_ in all of HF patients were below 2.0 ng/mL. The incidence of subtherapeutic steady-state through concentrations (C_min_ < 0.5 ng/mL) were 48.3% in class II, 52.8% in class III, and 63.3% in class IV HF patients under the optimized regimen. These values align with the corresponding incidence rates under the standard clinical regimen (0.25 mg once daily): 56.6% (class II), 56.0% (class III), and 59.4% (class IV). This study uses digoxin, a drug with a narrow therapeutic window, as an example. Its significant pharmacokinetic variability makes precise dosing crucial. Therefore, the optimization strategies and conclusions presented here are primarily applicable to digoxin and other drugs with similarly narrow therapeutic windows. For drugs with wider therapeutic windows, the primary focus of dose adjustment is typically on efficacy maximization rather than avoiding concentration-dependent toxicity; consequently, the reliance on precise dosing is relatively lower.

Unlike previous PBPK models of digoxin, which were primarily established in healthy populations and focused on predicting drug–drug interactions (such as those by Neuhoff et al. (2013) [55] and Moj et al. (2017) [61]), the present study develops a whole-body PBPK model for heart failure patients. Using digoxin as an example, we demonstrate the model’s utility in identifying optimal dosing strategies. This focus on HF pathophysiology reveals that the standard digoxin dose frequently results in subtherapeutic or toxic concentrations in these patients—a critical finding that could not be derived from previous models based on healthy physiology.

However, there are some limitations in the study. While the current model focuses on cardiac output, we acknowledge that it does not capture the full spectrum of inter patient heterogeneity in severe heart failure. Importantly, several critical patient characteristics were crucially omitted from the simulations, including age, gender, body weight, and drug–drug interactions. Furthermore, the absorption model employed in this study did not explicitly incorporate Biopharmaceutics Classification System parameters such as solubility; therefore, it may have limitations in predicting the absorption of poorly soluble drugs. In general, HF often occurs in elderly populations. Elderly populations are often associated with alterations in some physiological parameters, such as reductions in splanchnic blood flow, gastrointestinal motility, lean body mass, total body water, hepatic blood flow, hepatic mass, renal blood flow, and glomerular filtration rate, but also with increases in gastric pH, body fat, and 1-acid glycoprotein levels [62]. These alterations, in turn, influence pharmacokinetics of drugs in older populations. For example, elderly women often showed lower clearances of midazolam, triazolam, and verapamil than young women [63]. Gender also affects the pharmacokinetics of some drugs [63,64], as evidenced by reports that, women were reported to exhibit a higher clearance of midazolam than men [63,65]. In addition, it was assumed that HF only affected alterations in tissue blood flow and cardiac output without affecting other physiological parameters. A report [66] showed that patients with acute heart failure are associated with the thickening of the gastrointestinal wall and decreases in gastrointestinal motility, which may affect intestinal absorption. HF patients are often co-administered with many drugs, which leads to drug interactions.

## 5. Conclusions

A whole-body PBPK model was developed to predict drug disposition in HF patients across a range of disease severity. The model was applied to optimize the dosing regimen of digoxin as a case study.

## Figures and Tables

**Table 1:** Blood-flow rates of different tissues and organs in different degrees of HF (70 kg).

Heart Failure Class			
Blood Flow (mL/min)	II Grade	III Grade	IV Grade
Lung	4399.58	3610.12	3378.28
Muscle	427.5	330	210
Adipose	148.2	114.4	72.8
Skin	171	132	84
Kidney	967.2	682	781.2
Liver	228	162	138
Spleen	60.8	43.2	36.8
Stomach	28.88	20.52	17.48
Duodenum	89.68	63.72	54.28
Jejunum	313.88	223.02	189.98
Ileum	185.44	131.76	112.24
Cecum	33.44	23.76	20.24
Colon	213.56	151.74	129.26
Heart ^a^	240	240	240
Brain ^a^	700	700	700
ROB ^a^	592	592	592

^a^ With unchanged blood-flow velocity.

**Table 2:** Observed and predicted values of AUC_0-t_ and C_max_ of model drugs in HF patients.

Drug	Dose	Subjects	AUC_0-t_ (μg × h/mL)			C_max_ (ng/mL)		
			Obs	Pre	Obs/Pre	Obs	Pre	Obs/Pre
digoxin	0.1 mg ^a^ [48]	HF-IV	NR	0.0067	/	1.10	0.93	1.18
	0.25 mg ^b^ [49]	HF-III	0.024	0.020	1.2	1.72	2.46	0.70
furosemide	40 mg ^a^, i.v [32]	HF-III	7.98	5.78	1.38	/	/	/
	120 mg ^a^, i.v [21]	HF-IV	18.02	17.36	1.04	/	/	/
	40 mg ^a^ [50]	HF-II	3.96	3.92	1.01	1124	1057.70	1.06
	120 mg ^a^ [32]	HF-IV	10.99	11.78	0.93	NR	3173.23	/
bumetanide	3 mg ^a^ [29]	HF-IV	0.28	0.30	0.85	107	96.36	1.11
	3 mg,3 min ^a^, i.v [29]	HF-IV	0.33	0.33	1	/	/	/
torasemide	10 mg ^b^ [6]	HF-II	7.69	4.35	1.77	1159	1008.50	1.15
	10 mg ^c^ [32]	HF-II	4.80	4.35	1.1	1500	1008.50	1.49
captopril	25 mg [51]	HF-IV	NR	0.32	/	NR	248.68	/
valsartan	40 mg ^d^ [52]	HF-III	13.12	10.86	1.21	1940	1359.91	1.43
	80 mg ^d^ [52]	HF-III	25.94	21.72	1.19	3950	2719.87	1.45
	160 mg ^d^ [52]	HF-III	43.54	43.45	1	6400	5439.70	1.18
felodipine	5 mg ^d^ [53]	HF-II	0.016	0.013	1.23	4.07	2.65	1.54
	10 mg ^d^ [53]	HF-II	0.037	0.026	1.42	6.26	5.29	1.18
midazolam	7.5 mg ^a^ [5]	HF-IV	0.15	0.10	1.50	76	46.92	1.62

Specific time of AUC_0-t_: ^a^: AUC_0-∞_; ^b^: 24 h; ^c^: 36 h; ^d^: 12 h.

## Data Availability

Data is contained within the article.

## Supplementary Materials

The following supporting information can be downloaded at: https://www.mdpi.com/article/10.3390/pharmaceutics17111394/s1. Mass equations [67,68,69]; Description of the tested drugs [6,11,55,70,71,72,73,74,75,76,77,78,79]; Table S1: Physiological parameters used in the PBPK model in healthy subjects (70 kg) [80,81]; Table S2: Physicochemical parameters of drugs used for the PBPK model [5,9,10,29,31,55,56,57,74,75,79,81,82,83,84,85,86,87,88,89,90,91]; Table S3: Clinical information of drugs in the model [5,6,13,14,15,16,17,18,19,20,21,22,23,24,25,26,27,28,29,30,31,32,33,34,35,36,37,38,39,40,41,42,43,44,45,46,47]; Table S4: Observed and predicted values of AUC_0-t_ and C_max_ of model drugs in healthy subjects [5,6,21,29,32,48,49,50,51,52,53].

## Abbreviations

The following abbreviations are used in this manuscript:

HF	Heart failure
PBPK	Physiologically based pharmacokinetic
AUC	Area under the concentration-time curve
C_max_	Maximum plasma concentration
GSA	Global sensitivity analysis
NYHA	New York Heart Association
AUCR	The ratio of AUC in HF patients to that in healthy subjects
C_max_R	The ratio of C_max_ in HF patients to that in healthy subjects

## References

[1] New York Heart Association (1994). Nomenclature and Criteria for Diagnosis of Diseases of the Heart and Great Vessels.

[2] Leithe M.E., Margorien R.D., Hermiller J.B., Unverferth D.V., Leier C.V. (1984). Relationship between central hemodynamics and regional blood flow in normal subjects and in patients with congestive heart failure. Circulation.

[3] Moe G.W., Armstrong P.W. (1988). Congestive heart failure. Cmaj.

[4] Sandek A., Bauditz J., Swidsinski A., Buhner S., Weber-Eibel J., von Haehling S., Schroedl W., Karhausen T., Doehner W., Rauchhaus M. (2007). Altered intestinal function in patients with chronic heart failure. J. Am. Coll. Cardiol..

[5] Patel I.H., Soni P.P., Fukuda E.K., Smith D.F., Leier C.V., Boudoulas H. (1990). The pharmacokinetics of midazolam in patients with congestive heart failure. Br. J. Clin. Pharmacol..

[6] Kramer W.G. (1994). Pharmacokinetics and pharmacodynamics of torasemide in congestive heart failure. Cardiology.

[7] Sager J.E., Yu J., Ragueneau-Majlessi I., Isoherranen N. (2015). Physiologically Based Pharmacokinetic (PBPK) Modeling and Simulation Approaches: A Systematic Review of Published Models, Applications, and Model Verification. Drug Metab. Dispos..

[8] Zhuang X., Lu C. (2016). PBPK modeling and simulation in drug research and development. Acta Pharm. Sin. B.

[9] Rodgers T., Rowland M. (2006). Physiologically based pharmacokinetic modelling 2: Predicting the tissue distribution of acids, very weak bases, neutrals and zwitterions. J. Pharm. Sci..

[10] Ruark C.D., Hack C.E., Robinson P.J., Mahle D.A., Gearhart J.M. (2014). Predicting passive and active tissue:plasma partition coefficients: Interindividual and interspecies variability. J. Pharm. Sci..

[11] Rasool M.F., Ali S., Khalid S., Khalid R., Majeed A., Imran I., Saeed H., Usman M., Ali M., Alali A.S. (2021). Development and evaluation of physiologically based pharmacokinetic drug-disease models for predicting captopril pharmacokinetics in chronic diseases. Sci. Rep..

[12] Rasool M.F., Khalil F., Laer S. (2016). Predicting Stereoselective Disposition of Carvedilol in Adult and Pediatric Chronic Heart Failure Patients by Incorporating Pathophysiological Changes in Organ Blood Flows-A Physiologically Based Pharmacokinetic Approach. Drug Metab. Dispos..

[13] Eckermann G., Lahu G., Nassr N., Bethke T.D. (2012). Absence of pharmacokinetic interaction between roflumilast and digoxin in healthy adults. J. Clin. Pharmacol..

[14] Gurley B.J., Swain A., Williams D.K., Barone G., Battu S.K. (2008). Gauging the clinical significance of P-glycoprotein-mediated herb-drug interactions: Comparative effects of St. John’s wort, Echinacea, clarithromycin, and rifampin on digoxin pharmacokinetics. Mol. Nutr. Food Res..

[15] Martin P.D., Kemp J., Dane A.L., Warwick M.J., Schneck D.W. (2002). No effect of rosuvastatin on the pharmacokinetics of digoxin in healthy volunteers. J. Clin. Pharmacol..

[16] Verstuyft C., Strabach S., El-Morabet H., Kerb R., Brinkmann U., Dubert L., Jaillon P., Funck-Brentano C., Trugnan G., Becquemont L. (2003). Dipyridamole enhances digoxin bioavailability via P-glycoprotein inhibition. Clin. Pharmacol. Ther..

[17] Tarral A., Francheteau P., Guerret M. (1997). Effects of terbinafine on the pharmacokinetics of digoxin in healthy volunteers. Pharmacotherapy.

[18] Zhou H., Horowitz A., Ledford P.C., Hubert M., Appel-Dingemanse S., Osborne S., McLeod J.F. (2001). The effects of tegaserod (HTF 919) on the pharmacokinetics and pharmacodynamics of digoxin in healthy subjects. J. Clin. Pharmacol..

[19] Hayward R.P., Greenwood H., Hamer J. (1978). Comparison of digoxin and medigoxin in normal subjects. Br. J. Clin. Pharmacol..

[20] Smith D.E., Gee W.L., Brater D.C., Lin E.T., Benet L.Z. (1980). Preliminary evaluation of furosemide-probenecid interaction in humans. J. Pharm. Sci..

[21] Andreasen F., Mikkelsen E. (1977). Distribution, elimination and effect of furosemide in normal subjects and in patients with heart failure. Eur. J. Clin. Pharmacol..

[22] Keller E., Hoppe-Seyler G., Mumm R., Schollmeyer P. (1981). Influence of hepatic cirrhosis and end-stage renal disease on pharmacokinetics and pharmacodynamics of furosemide. Eur. J. Clin. Pharmacol..

[23] Hammarlund M.M., Paalzow L.K., Odlind B. (1984). Pharmacokinetics of furosemide in man after intravenous and oral administration. Application of moment analysis. Eur. J. Clin. Pharmacol..

[24] Andreasen F., Christensen C.K., Jacobsen F.K., Jansen J., Mogensen C.E., Pedersen O.L. (1982). The individual variation in pharmacokinetics and pharmacodynamics of furosemide in young normal male subjects. Eur. J. Clin. Investig..

[25] Verbeeck R.K., Patwardhan R.V., Villeneuve J.P., Wilkinson G.R., Branch R.A. (1982). Furosemide disposition in cirrhosis. Clin. Pharmacol. Ther..

[26] Kelly M.R., Cutler R.E., Forrey A.W., Kimpel B.M. (1974). Pharmacokinetics of orally administered furosemide. Clin. Pharmacol. Ther..

[27] Britz H., Hanke N., Taub M.E., Wang T., Prasad B., Fernandez É., Stopfer P., Nock V., Lehr T. (2020). Physiologically Based Pharmacokinetic Models of Probenecid and Furosemide to Predict Transporter Mediated Drug-Drug Interactions. Pharm. Res..

[28] Holazo A.A., Colburn W.A., Gustafson J.H., Young R.L., Parsonnet M. (1984). Pharmacokinetics of bumetanide following intravenous, intramuscular, and oral administrations to normal subjects. J. Pharm. Sci..

[29] Cook J.A., Smith D.E., Cornish L.A., Tankanow R.M., Nicklas J.M., Hyneck M.L. (1988). Kinetics, dynamics, and bioavailability of bumetanide in healthy subjects and patients with congestive heart failure. Clin. Pharmacol. Ther..

[30] Lau H.S., Hyneck M.L., Berardi R.R., Swartz R.D., Smith D.E. (1986). Kinetics, dynamics, and bioavailability of bumetanide in healthy subjects and patients with chronic renal failure. Clin. Pharmacol. Ther..

[31] Knauf H., Mutschler E. (1998). Clinical pharmacokinetics and pharmacodynamics of torasemide. Clin. Pharmacokinet..

[32] Vargo D.L., Kramer W.G., Black P.K., Smith W.B., Serpas T., Brater D.C. (1995). Bioavailability, pharmacokinetics, and pharmacodynamics of torsemide and furosemide in patients with congestive heart failure. Clin. Pharmacol. Ther..

[33] Duchin K.L., Singhvi S.M., Willard D.A., Migdalof B.H., McKinstry D.N. (1982). Captopril kinetics. Clin. Pharmacol. Ther..

[34] Chik Z., Basu R.C., Pendek R., Lee T.C., Mohamed Z. (2010). A bioequivalence comparison of two formulations of rifampicin (300- vs 150-mg capsules): An open-label, randomized, two-treatment, two-way crossover study in healthy volunteers. Clin. Ther..

[35] Jankowski A., Skorek A., Krzyśko K., Zarzycki P.K., Ochocka R.J., Lamparczyk H. (1995). Captopril: Determination in blood and pharmacokinetics after single oral dose. J. Pharm. Biomed. Anal..

[36] Kripalani K.J., McKinstry D.N., Singhvi S.M., Willard D.A., Vukovich R.A., Migdalof B.H. (1980). Disposition of captopril in normal subjects. Clin. Pharmacol. Ther..

[37] Singhvi S.M., McKinstry D.N., Shaw J.M., Willard D.A., Migdalof B.H. (1982). Effect of food on the bioavailability of captopril in healthy subjects. J. Clin. Pharmacol..

[38] Creasey W.A., Funke P.T., McKinstry D.N., Sugerman A.A. (1986). Pharmacokinetics of captopril in elderly healthy male volunteers. J. Clin. Pharmacol..

[39] Sioufi A., Marfil F., Jaouen A., Cardot J.M., Godbillon J., Ezzet F., Lloyd P. (1998). The effect of age on the pharmacokinetics of valsartan. Biopharm. Drug Dispos..

[40] Iqbal M., Khuroo A., Batolar L.S., Tandon M., Monif T., Sharma P.L. (2010). Pharmacokinetics and bioequivalence study of three oral formulations of valsartan 160 mg: A single-dose, randomized, open-label, three-period crossover comparison in healthy Indian male volunteers. Clin. Ther..

[41] Landahl S., Edgar B., Gabrielsson M., Larsson M., Lernfelt B., Lundborg P., Regårdh C.G. (1988). Pharmacokinetics and blood pressure effects of felodipine in elderly hypertensive patients. A comparison with young healthy subjects. Clin. Pharmacokinet..

[42] Bailey D.G., Arnold J.M., Munoz C., Spence J.D. (1993). Grapefruit juice--felodipine interaction: Mechanism, predictability, and effect of naringin. Clin. Pharmacol. Ther..

[43] Larsson R., Karlberg B.E., Gelin A., Aberg J., Regårdh C.G. (1990). Acute and steady-state pharmacokinetics and antihypertensive effects of felodipine in patients with normal and impaired renal function. J. Clin. Pharmacol..

[44] Hardy B.G., Bartle W.R., Myers M., Bailey D.G., Edgar B. (1988). Effect of indomethacin on the pharmacokinetics and pharmacodynamics of felodipine. Br. J. Clin. Pharmacol..

[45] Kharasch E.D., Francis A., London A., Frey K., Kim T., Blood J. (2011). Sensitivity of intravenous and oral alfentanil and pupillary miosis as minimal and noninvasive probes for hepatic and first-pass CYP3A induction. Clin. Pharmacol. Ther..

[46] Ahonen J., Olkkola K.T., Neuvonen P.J. (1995). Effect of itraconazole and terbinafine on the pharmacokinetics and pharmacodynamics of midazolam in healthy volunteers. Br. J. Clin. Pharmacol..

[47] Abdlekawy K.S., Donia A.M., Elbarbry F. (2017). Effects of Grapefruit and Pomegranate Juices on the Pharmacokinetic Properties of Dapoxetine and Midazolam in Healthy Subjects. Eur. J. Drug Metab. Pharmacokinet..

[48] Ohnhaus E.E., Vozeh S., Nuesch E. (1979). Absorption of digoxin in severe right heart failure. Eur. J. Clin. Pharmacol..

[49] Liu Z., Fang S., Wang L., Zhu T., Yang H., Yu S. (1998). Clinical study on chronopharmacokinetics of digoxin in patients with congestive heart failure. Curr. Med. Sci..

[50] Müller F.O., Middle M.V., Schall R., Terblanché J., Hundt H.K., Groenewoud G. (1997). An evaluation of the interaction of meloxicam with frusemide in patients with compensated chronic cardiac failure. Br. J. Clin. Pharmacol..

[51] Shaw T.R., Duncan F.M., Williams B.C., Crichton E., Thomson S.A., Davis J.R., Rademaker M., Edwards C.R. (1985). Plasma free captopril concentrations during short and long term treatment with oral captopril for heart failure. Br. Heart J..

[52] Prasad P.P., Yeh C.M., Gurrieri P., Glazer R., McLeod J. (2002). Pharmacokinetics of multiple doses of valsartan in patients with heart failure. J. Cardiovasc. Pharmacol..

[53] Rehnqvist N., Billing E., Moberg L., Lundman T., Olsson G. (1987). Pharmacokinetics of felodipine and effect on digoxin plasma levels in patients with heart failure. Drugs.

[54] Guo Z., Gao J., Liu L., Liu X. (2024). Quantitatively Predicting Effects of Exercise on Pharmacokinetics of Drugs Using a Physiologically Based Pharmacokinetic Model. Drug Metab. Dispos..

[55] Neuhoff S., Yeo K.R., Barter Z., Jamei M., Turner D.B., Rostami-Hodjegan A. (2013). Application of permeability-limited physiologically-based pharmacokinetic models: Part I-digoxin pharmacokinetics incorporating P-glycoprotein-mediated efflux. J. Pharm. Sci..

[56] Tsutsumi K., Kotegawa T., Kuranari M., Otani Y., Morimoto T., Matsuki S., Nakano S. (2002). The effect of erythromycin and clarithromycin on the pharmacokinetics of intravenous digoxin in healthy volunteers. J. Clin. Pharmacol..

[57] Qian C.Q., Zhao K.J., Chen Y., Liu L., Liu X.D. (2019). Simultaneously predict pharmacokinetic interaction of rifampicin with oral versus intravenous substrates of cytochrome P450 3A/P-glycoprotein to healthy human using a semi-physiologically based pharmacokinetic model involving both enzyme and transporter turnover. Eur. J. Pharm. Sci..

[58] See I., Shehab N., Kegler S.R., Laskar S.R., Budnitz D.S. (2014). Emergency department visits and hospitalizations for digoxin toxicity: United States, 2005 to 2010. Circ. Heart Fail..

[59] Ordog G.J., Benaron S., Bhasin V., Wasserberger J., Balasubramanium S. (1987). Serum digoxin levels and mortality in 5,100 patients. Ann. Emerg. Med..

[60] Yancy C.W., Jessup M., Bozkurt B., Butler J., Casey D.E., Drazner M.H., Fonarow G.C., Geraci S.A., Horwich T., Januzzi J.L. (2013). 2013 ACCF/AHA guideline for the management of heart failure: A report of the American College of Cardiology Foundation/American Heart Association Task Force on practice guidelines. Circulation.

[61] Moj D., Hanke N., Britz H., Frechen S., Kanacher T., Wendl T., Haefeli W.E., Lehr T. (2017). Clarithromycin, Midazolam, and Digoxin: Application of PBPK Modeling to Gain New Insights into Drug-Drug Interactions and Co-medication Regimens. AAPS J..

[62] Klotz U. (2009). Pharmacokinetics and drug metabolism in the elderly. Drug Metab. Rev..

[63] Cotreau M.M., von Moltke L.L., Greenblatt D.J. (2005). The influence of age and sex on the clearance of cytochrome P450 3A substrates. Clin. Pharmacokinet..

[64] Wilson K. (1984). Sex-related differences in drug disposition in man. Clin. Pharmacokinet..

[65] Hu Z.Y., Zhao Y.S. (2010). Sex-dependent differences in cytochrome P450 3A activity as assessed by midazolam disposition in humans: A meta-analysis. Drug Metab. Dispos..

[66] Hao R., Zheng Y., Zhao Q., Chen J., Fan R., Chen P., Yin N., Qin H. (2024). Evaluation value of ultrasound on gastrointestinal function in patients with acute heart failure. Front. Cardiovasc. Med..

[67] Edginton A.N., Willmann S. (2008). Physiology-based simulations of a pathological condition: Prediction of pharmacokinetics in patients with liver cirrhosis. Clin. Pharmacokinet..

[68] Yu L.X., Amidon G.L. (1999). A compartmental absorption and transit model for estimating oral drug absorption. Int. J. Pharm..

[69] Gertz M., Houston J.B., Galetin A. (2011). Physiologically based pharmacokinetic modeling of intestinal first-pass metabolism of CYP3A substrates with high intestinal extraction. Drug Metab. Dispos..

[70] Scalese M.J., Salvatore D.J. (2017). Role of Digoxin in Atrial Fibrillation. J. Pharm. Pract..

[71] Lainscak M., Vitale C., Seferovic P., Spoletini I., Cvan Trobec K., Rosano G.M. (2016). Pharmacokinetics and pharmacodynamics of cardiovascular drugs in chronic heart failure. Int. J. Cardiol..

[72] Solanki D., Choudhary S., Vora A., Ghose T., Mantri R.R., Modi N., Sawhney J., Singhal A., Kumar A., Edakutty R. (2024). Loop Diuretics Unique Mechanism of Action. J. Assoc. Physicians India.

[73] Ponto L.L., Schoenwald R.D. (1990). Furosemide (frusemide). A pharmacokinetic/pharmacodynamic review (Part I). Clin. Pharmacokinet..

[74] Chapa R., Li C.Y., Basit A., Thakur A., Ladumor M.K., Sharma S., Singh S., Selen A., Prasad B. (2020). Contribution of Uptake and Efflux Transporters to Oral Pharmacokinetics of Furosemide. ACS Omega.

[75] Donovan M.D., Abduljalil K., Cryan J.F., Boylan G.B., Griffin B.T. (2018). Application of a physiologically-based pharmacokinetic model for the prediction of bumetanide plasma and brain concentrations in the neonate. Biopharm. Drug Dispos..

[76] Brogden R.N., Todd P.A., Sorkin E.M. (1988). Captopril. An update of its pharmacodynamic and pharmacokinetic properties, and therapeutic use in hypertension and congestive heart failure. Drugs.

[77] Michel M.C., Foster C., Brunner H.R., Liu L. (2013). A systematic comparison of the properties of clinically used angiotensin II type 1 receptor antagonists. Pharmacol. Rev..

[78] Yedinak K.C., Lopez L.M. (1991). Felodipine: A new dihydropyridine calcium-channel antagonist. Dicp.

[79] Salem F., Nimavardi A., Mudunuru J., Tompson D., Bloomer J., Turner D.B., Taskar K.S. (2023). Physiologically based pharmacokinetic modeling for development and applications of a virtual celiac disease population using felodipine as a model drug. CPT Pharmacomet. Syst. Pharmacol..

[80] Kong W.M., Sun B.B., Wang Z.J., Zheng X.K., Zhao K.J., Chen Y., Zhang J.X., Liu P.H., Zhu L., Xu R.J. (2020). Physiologically based pharmacokinetic-pharmacodynamic modeling for prediction of vonoprazan pharmacokinetics and its inhibition on gastric acid secretion following intravenous/oral administration to rats, dogs and humans. Acta Pharmacol. Sin..

[81] Yang Y., Li P., Zhang Z., Wang Z., Liu L., Liu X. (2020). Prediction of Cyclosporin-Mediated Drug Interaction Using Physiologically Based Pharmacokinetic Model Characterizing Interplay of Drug Transporters and Enzymes. Int. J. Mol. Sci..

[82] Jeong S.H., Jang J.H., Lee Y.B. (2022). Torsemide Pharmacometrics in Healthy Adult Populations Including CYP2C9 Genetic Polymorphisms and Various Patient Groups through Physiologically Based Pharmacokinetic-Pharmacodynamic Modeling. Pharmaceutics.

[83] McPherson S., Perrier J., Dunn C., Khadra I., Davidson S., Ainousah B., Wilson C.G., Halbert G. (2020). Small scale design of experiment investigation of equilibrium solubility in simulated fasted and fed intestinal fluid. Eur. J. Pharm. Biopharm..

[84] Fuhr L.M., Marok F.Z., Mees M., Mahfoud F., Selzer D., Lehr T. (2022). A Physiologically Based Pharmacokinetic and Pharmacodynamic Model of the CYP3A4 Substrate Felodipine for Drug-Drug Interaction Modeling. Pharmaceutics.

[85] Schlender J.F., Meyer M., Thelen K., Krauss M., Willmann S., Eissing T., Jaehde U. (2016). Development of a Whole-Body Physiologically Based Pharmacokinetic Approach to Assess the Pharmacokinetics of Drugs in Elderly Individuals. Clin. Pharmacokinet..

[86] Poirier A., Cascais A.C., Funk C., Lavé T. (2009). Prediction of pharmacokinetic profile of valsartan in human based on in vitro uptake transport data. J. Pharmacokinet. Pharmacodyn..

[87] Pandey M.M., Jaipal A., Kumar A., Malik R., Charde S.Y. (2013). Determination of pK(a) of felodipine using UV-Visible spectroscopy. Spectrochim. Acta A Mol. Biomol. Spectrosc..

[88] Hanke N., Frechen S., Moj D., Britz H., Eissing T., Wendl T., Lehr T. (2018). PBPK Models for CYP3A4 and P-gp DDI Prediction: A Modeling Network of Rifampicin, Itraconazole, Clarithromycin, Midazolam, Alfentanil, and Digoxin. CPT Pharmacomet. Syst. Pharmacol..

[89] Schwartz S., Brater D.C., Pound D., Green P.K., Kramer W.G., Rudy D. (1993). Bioavailability, pharmacokinetics, and pharmacodynamics of torsemide in patients with cirrhosis. Clin. Pharmacol. Ther..

[90] Flesch G., Müller P., Lloyd P. (1997). Absolute bioavailability and pharmacokinetics of valsartan, an angiotensin II receptor antagonist, in man. Eur. J. Clin. Pharmacol..

[91] Rowland Yeo K., Walsky R.L., Jamei M., Rostami-Hodjegan A., Tucker G.T. (2011). Prediction of time-dependent CYP3A4 drug-drug interactions by physiologically based pharmacokinetic modelling: Impact of inactivation parameters and enzyme turnover. Eur. J. Pharm. Sci..

