# Supplementary material for: Predicting Pharmacokinetics of Drugs in Patients with Heart Failure and Optimizing Their Dosing Strategies Using a Physiologically Based Pharmacokinetic Model
Source: Pharmaceutics. 2025 Oct 28;17(11):1394. doi: 10.3390/pharmaceutics17111394 (PMC12655055; doi:10.3390/pharmaceutics17111394)
Supplement: Supplementary file 1 [file pharmaceutics-17-01394-s001.zip › pharmaceutics-3855422-supplementary.pdf]

pharmaceutics

MDPI

Supplementary Materials: Predicting Pharmacokinetics of Drugs in Patients with Heart Failure and Optimizing Their Dosing strategies Using a Physiologically Based Pharmacokinetic Model

Weiye Gu, Qingxuan Shao, Ling Jiang*

1. Mass Equations

Drug concentrations in corresponding compartment was illustrated as follows.

For general tissues (except lung) compartments (t):

$$V_t \frac{dC_t}{dt} = Q_t \times \left(C_a - \frac{C_t}{K_{t:p}/R_b}\right) \tag{S1}$$

where V_t denotes tissue volume, C_t represents drug concentration in tissues, and Q_t refers to tissue blood flow. C_a corresponds to the drug concentration in arterial blood. $K_{t:p}$ and R_b are ratios of drug concentration in tissues to plasma and in blood to plasma, separately.

For venous blood (v):

$$V_v \frac{dC_v}{dt} = \sum\left(Q_t \times \frac{C_t}{K_{t:p}/R_b}\right) - Q_{total} \times C_v \tag{S2}$$

For lung (lu):

$$V_{lu} \frac{dC_{lu}}{dt} = Q_{total} \times \left(C_v - \frac{C_{lu}}{K_{lu:p}/R_b}\right) \tag{S3}$$

For arterial blood (a):

$$V_a \frac{dC_a}{dt} = Q_{total} \times \left(\frac{C_{lu}}{K_{lu:p}/R_b} - C_a\right) \tag{S4}$$

Where Q_{total} is cardiac output.

For hepatic compartment (li):

$$V_{li} \frac{dC_{li}}{dt} = Q_{la} \times C_a + \frac{C_{st} \times Q_{st}}{K_{st:p}/R_b} + \frac{C_{sp} \times Q_{sp}}{K_{sp:p}/R_b} + \sum \frac{C_{Gwi} \times Q_{Gwi}}{K_{g:p}/R_b} - (Q_{Li} + f_{u,b} CL_{li,int}) \frac{C_{li}}{K_{li:p}/R_b} \tag{S5}$$

Where Q_{li}, Q_{la}, Q_{st}, Q_{sp} and Q_{Gwi} are respectively blood flows in liver, artery, stomach, spleen and the ith intestinal wall. $Q_{li} = Q_{la} + Q_{st} + Q_{sp} + \sum Q_{Gwi}$. $CL_{li,int}$ and $f_{u,b}$ represent the hepatic intrinsic clearance of drug and the free fraction of drug in blood, respectively. The blood free fraction ($f_{u,b}$) is calculated from the plasma free fraction ($f_{u,p}$) using equation S6.

$$f_{u,b} = \frac{f_{u,p}}{R_b} \tag{S6}$$

$CL_{li,int}$ is recalculated from hepatic clearance of drug ($CL_{li,b}$) using equation S7.

$$CL_{li,b} = \frac{Q_{Li} \times f_{u,b} \times CL_{li,int}}{Q_{Li} + f_{u,b} \times CL_{li,int}} \tag{S7}$$

$CL_{li,b}$ may be derived from the total clearance (CL_{tol}) and the renal clearance (CL_K), i.e.

$$CL_{li} = CL_{tol} - CL_K \tag{S8}$$

In clinical practice, the reported clearance is often plasma clearance (CL_P), thus CL_b is estimated using equation S9.

$$CL_b = \frac{CL_p}{1 - Hct + R_b \times Hct} \tag{S9}$$

Where Hct is hematocrit, which is set to be 0.43 [1].

In kidney (k):

$$V_k \frac{dC_k}{dt} = Q_k \times C_a - (Q_k + f_{ub} \times CL_{k,int}) \times \frac{C_k}{K_{k:p}/R_b} \quad (S10)$$

$CL_{K,int}$ is recalculated from renal clearance of drug (CL_K) using equation S11.

$$CL_K = \frac{Q_K \times f_{u,b} \times CL_{K,int}}{Q_K + f_{u,b} \times CL_{K,int}} \quad (S11)$$

For oral administration:

Gastrointestinal tract is divided into stomach, duodenum, jejunum, ileum, caecum and colon. It is assumed that absorption of drug only occurs in duodenum, jejunum and ileum. Drug concentrations in intestinal lumen and wall are illustrated as follows.

In stomach (A_0):

$$\frac{dA_0}{d_t} = -K_{t,0} \times A_0 \quad (S12)$$

Where $K_{t,0}$ is constant of gastric emptying rate.

In intestinal lumen (A_i):

$$\frac{dA_i}{d_t} = K_{t,i-1} \times A_{i-1} - (K_{t,i-1} + k_{a,i}) \times A_i \quad (S13)$$

In intestinal wall (A_{Gwi}):

$$\frac{dA_{Gwi}}{d_t} = Q_{Gwi} \times C_a + k_{a,i} \times A_i - (Q_{Gwi} + f_{u,g} \times CL_{Gwi,int}) \times \frac{A_{Gwi}/V_{Gwi}}{K_{g:p}/R_b} \quad (S14)$$

Where $K_{t,i}$ and $k_{a,i}$ are respectively constants of intestinal transit rate and intestinal absorption rate. Q_{Gwi} and V_{Gwi} are separately blood flow in the ith intestine wall and volume of the ith intestine wall (i = duodenum, jejunum and ileum). $k_{a,i}$ may estimate using a set of plasma concentration-time profile on WinNonlin 8.4 or derived from effective permeability coefficient ($P_{eff,A-B}$) using equation S15.

$$k_{a,i} = \frac{2 \times P_{eff,A-B}}{r_i} \quad (S15)$$

Where r_i is the intestinal radius. $CL_{gwi,int}$ and $f_{u.g}$ are the intrinsic clearance and free fraction of drug in the ith intestinal wall, respectively. $f_{u,g}$ was assumed to be 1 for oral administration [2]. If metabolism of the tested drugs in the intestine is mainly mediated by CYP3A, the $CL_{gwi,int}$ of the tested drugs in the ith intestinal segment can be extrapolated from hepatic metabolic parameters using a scaling factor (i.e, ratio of CYP3A amount in intestine to liver). The amount of CYP3A in the duodenum, jejunum, and ileum was set to be 9.7 nmol, 38.4 nmol, and 22.4 nmol [3], respectively. Amount of hepatic CYP3A was set to be 155 pmol/mg hepatic microsome protein (12555 nmol / 70 kg man) [3].

2. Description of the tested drugs

2.1. Digoxin

Digoxin is a medium-acting cardiac glycoside drug. Oral absorption of digoxin is incomplete and irregular [4]. Digoxin is eliminated primarily through kidney [5]. The free fraction in the plasma is 0.71 [6]. Nine clinical reports, including two reports involving HF, were selected in the simulations.

2.2. Furosemide

Furosemide is a potent diuretic that is primarily excreted by the kidneys, with a small amount also being eliminated via bile through liver metabolism [7,8]. Furosemide shows high plasma protein binding, whose free fraction in plasma is only 0.03 [9]. Ten clinical reports, including three reports involving different grades of HF, were selected in the simulations.

2.3. Bumetanide

Bumetanide is a potent diuretic that is eliminated via renal excretion. It belongs to drugs with high plasma binding, whose free fraction in plasma is 0.03 [10]. Three clinical reports, including one report involving HF, were selected in the simulations.

2.4. Torsemide

Torsemide, a potent loop diuretic, demonstrates 20% renal excretion, with the remaining 80% undergoing hepatic metabolism prior to systemic elimination [11]. The free fraction of torsemide in plasma is 0.01 [12]. Three clinical reports, including two reports involving HF, were selected in the simulations.

2.5. Captopril

Captopril is an angiotensin converting enzyme inhibitor, which mainly acts on the renin-angiotensin-aldosterone system. It undergoes predominant hepatic metabolism, with renal excretion of both metabolites and 40-50% of the unchanged drug [5,13]. The free fraction of captopril in plasma is 0.73 [14]. Seven clinical reports, including one report involving HF, were selected in the simulations.

2.6. Valsartan

Valsartan, an angiotensin II receptor antagonist, is eliminated via biliary and renal excretion [15]. The free fraction of valsartan in plasma is 0.05 [16]. Three clinical reports, including one report involving HF, were selected in the simulations.

2.7. Felodipine

Felodipine is a calcium channel blocker, it can significantly reduce systolic and diastolic blood pressure in patients with hypertension [17]. Its metabolism is predominantly mediated by hepatic and intestinal CYP3A. The free fraction of felodipine in plasma is 0.0048 [18]. Five clinical reports, including one report involving HF, were selected in the simulations.

2.8. Midazolam

Midazolam is a short-acting benzodiazepine derivative. Although it has no direct therapeutic effect on HF, it can be used in acute episodes of HF. Midazolam can rapidly alleviate anxiety, reduce sympathetic nervous system activity, and decrease catecholamine release, thereby lowering myocardial oxygen consumption and improving cardiac load. Metabolism of midazolam is predominantly mediated by hepatic and intestinal CYP3A. The free fraction of midazolam in plasma is 0.031 [3]. Four clinical reports, including one report involving HF, were selected in the simulations.

3. Tables

Table S1. Physiological parameters used in the PBPK model in healthy subjects (70 kg).

Tissue	Volume [19] (mL)	Blood flow [20] (mL/min)	Transit rate constant [20] (min^{-1})	Intestinal radius [19] (cm)
Lung	1170	5600	/	/
Kidney	280	1240	/	/
Heart	310	240	/	/
Liver	1690	1518[a]	/	/
Muscle	35000	750	/	/
Skin	7800	300	/	/
Brain	1450	700	/	/
Adipose	10000	260	/	/

ROB	5100	592	/	/
Spleen	190	80	/	/
Venous	3470	/	/	/
Artery	1730	/	/	/
Stomach	160	38	0.0462	/
Duodenum	70	118	0.0462	2
Jejunum	209	413	0.012	1.63
Ileum	139	244	0.0058	1.45
Cecum	116	44	0.0025	/
Colon	1116	281	0.00085	/

[a]: It is assumed that the hepatic blood flow is the sum of the blood flow of hepatic artery, spleen, stomach and intestine.

Table S2. Physicochemical parameters of drugs used for the PBPK model.

	**Digoxin**	**Furosemide**	**Bumetanide**	**Torasemide**	**Captopril**	**Valsartan**	**Felodipine**	**Midazolam**
$\log_P$	2.37[a]	2.29 [9]	3.6 [10]	0.57 [21]	0.34 [14]	1.5 [22]	4.36 [23]	2.89 [24]
pka	13.5 [24]	4.25 [25]	2.6 [10]	7.1 [21]	4.02 [14]	4.73 [26]	5.07 [27]	6.15 [28]
$f_{u,plasma}$	0.71 [6]	0.03 [9]	0.03 [10]	0.01 [12]	0.73 [14]	0.05 [26]	0.0048 [18]	0.031 [20]
R_{bp}	1[c]	0.5 [9]	0.55 [10]	1[c]	1 [14]	1[c]	0.7 [18]	0.55 [20]
$P_{eff,A\text{-}B}$ (cm/min)	0.0282[6]	0.013[9]	/	/	/	/	/	0.04 [20]
CL_{renal} (mL/min)	98.4 [29]	112.7 [25]	146 [30]	6.4 [31]	370 [14]	10.3 [32]	27.6 [33]	1.42 [34]
CL_{liver} (mL/min)	41.6 [29]	53.9 [25]	62 [30]	36.6 [31]	455 [14]	26.2 [32]	795.7 [18]	546.8 [35]
$CL_{GW1,int}$ (mL/min)	/	/	/	/	/	/	256.6[d]	26.8[d]
$CL_{GW2,int}$ (mL/min)	/	/	/	/	/	/	1016[d]	106.3[d]
$CL_{GW3,int}$ (mL/min)	/	/	/	/	/	/	592.7[d]	62[d]
k_a (1/min)	/	/	0.014[b]	0.07[b]	0.099[b]	0.008[b]	0.0032[b]	/
$f_{u,g}$	1[c]	1[c]	1[c]	1[c]	1[c]	1[c]	0.12[18]	1[c]
$K_{Adipose:plasma}$	142.15[e]	0.055[f]	0.05[e]	0.028[f]	0.39[f]	0.045[f]	2.21[f]	3.89[f]
$K_{Liver:plasma}$	10.83[24]	0.29[f]	0.1[e]	0.015[f]	0.97[f]	0.14[f]	5.66[f]	1.37[f]
$K_{Muscle: plasma}$	7.35[36]	0.22[f]	0.07[e]	0.02[f]	1.32[f]	0.19[f]	4.69[f]	1.27[f]
$K_{Lung: plasma}$	4.83[e]	0.49[f]	0.22[e]	0.027[f]	1.85[f]	0.33[f]	13.18[f]	3.79[f]
$K_{Kidney: plasma}$	3.73[e]	0.26[f]	0.14[e]	0.016[f]	0.99[f]	0.14[f]	4.91[f]	1.2[f]
$K_{stomach: plasma}$	2.55[e]	0.24[f]	0.07[e]	0.019[f]	10.08[f]	0.16[f]	5.14[f]	1.52[f]
$K_{ROB: plasma}$	0.01[e]	0.01[f]	0.01[e]	0.01[f]	0.01[f]	0.01[f]	0.01[f]	0.01[f]
$K_{Brain:plasma}$	7.11[e]	0.094[f]	0.06[e]	0.019[f]	1.08[f]	0.1[f]	5.14[f]	0.75[f]
$K_{Heart: plasma}$	3.42[e]	0.18[f]	0.17[e]	0.017[f]	1.04[f]	0.12[f]	0.01[f]	0.86[f]
$K_{Spleen:plasma}$	2.36[e]	0.25[f]	0.11[e]	0.019[f]	1.2[f]	0.16[f]	1.31[f]	1.31[f]
$K_{Skin:plasma}$	10.64[e]	0.24[f]	0.28[e]	0.019[f]	1.2[f]	0.14[f]	3.23[f]	1.09[f]
$K_{gut:plasma}$	7.88[36]	0.24[f]	0.07[e]	0.019[f]	1.08[f]	0.16[f]	5.16[f]	1.52[f]

[a]: Data from www.drugbank.com; [b]: Simulation by WinNonlin; [c]: Assumed values; [d]: Specific activity based on liver metabolic parameters, Gw1-3 respectively represent Duodenal, Jejunal and Ileal. [e]: estimated using a method previously reported [37]; [f]: estimated using a method previously reported [38].

Table S3. Clinical information of drugs in the model.

No	Authors	Drug	Dose (mg)	Age	Subjects (n)	Ref
1	Eckermann G et al., 2012	digoxin	0.25, p.o	18-45	Healthy (16)	[39]
2	Gurley BJ et al., 2008	digoxin	0.25, p.o	30±5.4	Healthy (18)	[40]
3	Martin PD et al., 2002	digoxin	0.5, p.o	31-50	Healthy (18)	[41]
4	Verstuyft C et al., 2003	digoxin	0.5, p.o	22-35	Healthy (12)	[42]
5	Tarra A et al., 1997	digoxin	0.75, p.o	21-34	Healthy (16)	[43]
6	Zhou H et al., 2001	digoxin	1, p.o	25±3	Healthy (12)	[44]
7	Hayward RP et al., 1993	digoxin	1, p.o	23-32	Healthy (5)	[45]
8	Ohnhaus EE et al., 1979	digoxin	0.1, p.o	/	HF-IV(8)	[46]
9	Liu Z et al., 1998	digoxin	0.25, p.o	25-67	HF-III (10)	[47]
10	Britz et al., 2020	furosemide	20, p.o	/	Healthy (/)	[48]
11	Andreasen F et al., 1982	furosemide	80, i.v	25-35	Healthy (7)	[49]
12	Verbeeck RK et al., 1982	furosemide	80, i.v	23-42	Healthy (10)	[50]
13	Kelly MR et al., 1974	furosemide	80, i.v	18-45	Healthy (4)	[51]
14	Smith DE et al., 1980	furosemide	40, i.v	21-33	Healthy (4)	[52]
15	Keller E et al., 1981	furosemide	40, i.v	20-45	Healthy (7)	[53]
16	Hammarlund MM et al., 1984	furosemide	40, i.v	20-32	Healthy (8)	[54]
17	Andreasen F et al., 1977	furosemide	40, i.v	27-74	Healthy (8)	[55]
18	Andreasen F et al., 1977	furosemide	40, i.v	40-90	HF-III (26)	[55]
19	Müller FO et al., 1997	furosemide	40, p.o	50-74	HF-II (19)	[56]
20	Vargo DL et al., 1995	furosemide	120, i.v	45-72	HF-IV (7)	[57]
21	Vargo DL et al., 1995	furosemide	120, p.o	45-72	HF-IV (7)	[57]
22	Holazo AA et al., 1984	bumetanide	1, p.o	/	Healthy (12)	[58]
23	Holazo AA et al., 1984	bumetanide	1, i.v	/	Healthy (12)	[58]
24	Cook JA et al., 1988	bumetanide	3, p.o	23-38	Healthy (4)	[30]
25	Cook JA et al., 1988	bumetanide	3/3min, i.v	23-38	Healthy (4)	[30]
26	Cook JA et al., 1988	bumetanide	3, p.o	38-71	HF-IV (6)	[30]
27	Cook JA et al., 1988	bumetanide	3/3min, i.v	38-71	HF-IV (6)	[30]
28	Lau HS et al., 1986	bumetanide	5, p.o	22-32	Healthy (4)	[59]
29	Lau HS et al., 1986	bumetanide	5/3min, i.v	22-32	Healthy (4)	[59]

Table S3. Clinical information of drugs in the model (cont.).

No	Authors	Drug	Dose (mg)	Age	Subjects (n)	Ref
30	Knauf H et al.,1998	torasemide	5, p.o	22-27	Healthy (12)	[12]
31	Knauf H et al.,1998	torasemide	5, p.o	65-83	Healthy (11)	[12]
32	Kramer WG et al.,1994	torasemide	10, p.o	/	Healthy (/)	[11]
33	Vargo DL et al., 1995	torasemide	10, p.o	/	Healthy (/)	[57]
34	Kramer WG et al.,1994	torasemide	10, p.o	/	HF-II (/)	[11]
35	Vargo DL et al., 1995	torasemide	10, p.o	39-81	HF-II (16)	[57]
36	Duchin KL et al., 1982	captopril	10, p.o	24-34	Healthy (5)	[60]
37	Chik et al., 2010	captopril	25, p.o	19-25	Healthy (24)	[61]
38	Jankowski A et al., 1995	captopril	25, p.o	20-24	Healthy (12)	[62]
39	Kripalani KJ et al., 1980	captopril	100, p.o	18-35	Healthy (10)	[63]
40	Singhvi S M et al., 1982	captopril	100, p.o	18-33	Healthy (12)	[64]
41	Creasey WA et al., 1986	captopril	100, p.o	65-76	Healthy (12)	[65]
42	Shaw TR et al., 1985	captopril	25, p.o	47-71	HF-IV (20)	[66]
37	Sioufi A et al., 1998	valsartan	80, p.o	65-89	Healthy (12)	[67]
38	Iqbal et al., 2010	valsartan	160, p.o	20-34	Healthy (18)	[68]
39	Prasad et al., 2002	valsartan	40, p.o	18-75	HF-III (18)	[69]
40	Prasad et al., 2002	valsartan	80, p.o	18-75	HF-III (18)	[69]
41	Prasad et al., 2002	valsartan	160, p.o	18-75	HF-III (18)	[69]
42	Landahl S et al., 1988	felodipine	5, p.o	20-34	Healthy (12)	[70]
43	Bailey DG et al., 1993	felodipine	5, p.o	19-40	Healthy (9)	[71]
44	Larsson R et al., 1990	felodipine	10, p.o	36-74	Healthy (18)	[33]
45	Hardy BG et al., 1988	felodipine	10, p.o	22-39	Healthy (12)	[72]
46	Rehnqvist N et al., 1987	felodipine	5, p.o	59-68	HF-II (6)	[73]
47	Rehnqvist N et al., 1987	felodipine	10, p.o	59-68	HF-II (12)	[73]
48	Kharasch E D et al., 2011	midazolam	3, p.o	/	Healthy (12)	[74]
49	Patel I H et al., 1990	midazolam	7.5, p.o	36-54	Healthy (6)	[35]
50	Ahonen J et al.,1995	midazolam	7.5, p.o	19-30	Healthy (12)	[75]
51	Abdlekawy K S et al.,2017	midazolam	7.5, p.o	/	Healthy (12)	[76]
52	Patel I H et al., 1990	midazolam	7.5, p.o	38-61	HF-IV (6)	[35]

Table S4. Observed and predicted values of AUC_{0-t} and C_{max} of model drugs in healthy subjects.

Drug	Dose	AUC_{0-t} (μg × h/mL)			C_{max} (ng/mL)		
		Obs	Pre	Obs/Pre	Obs	Pre	Obs/Pre
digoxin	0.25 mg[a] [39]	0.017	0.013	1.31	1.51	1.43	1.06
	0.25 mg[b] [40]	0.0073	0.0077	0.95	1.20	1.41	0.85
	0.5 mg[c] [41]	0.0085	0.010	0.85	2.21	2.85	0.78
	0.5 mg[d] [42]	0.027	0.022	1.23	3.00	2.85	1.05
	0.75 mg[e] [43]	0.027	0.053	0.51	4.14	3.64	1.14
	1 mg[e] [44]	0.075	0.070	1.07	4.80	4.85	0.99
	1 mg[a] [45]	0.15	0.13	1.15	NR	4.85	/
furosemide	20mg [48]	NR	1.60	/	NR	524.57	/
	80mg[a],i.v. [49]	8.20	9.78	0.84	/	/	/
	80mg[a],i.v. [50]	8.55	9.78	0.87	/	/	/
	80mg[a],i.v. [51]	9.66	9.78	0.99	/	/	/
	40mg[a],i.v. [52]	4.20	4.66	0.90	/	/	/
	40mg[a],i.v. [53]	3.83	4.66	0.82	/	/	/
	40mg[a],i.v. [54]	4.23	4.66	0.91	/	/	/
	40mg[a],i.v. [55]	4.02	4.66	0.86	/	/	/
bumetanide	1mg[a] [58]	0.066	0.10	0.66	30.9	32.9	0.94

	3mg[a] [30]	0.20	0.30	0.67	106	98.8	1.07
	5mg[a] [59]	0.35	0.50	0.70	/	/	/
	1mg,2min[a],i.v. [58]	0.089	0.13	0.68	/	/	/
	3mg,3min[a],i.v. [30]	0.29	0.35	0.83	/	/	/
	5mg,3min[a],i.v. [59]	0.59	0.68	0.87	/	/	/
torasemide	5mg[a] [12]	1.77	1.89	0.94	535	508	1.05
	5mg[a] [12]	1.71	1.89	0.90	552	508	1.09
	10mg[f] [11]	3.67	4.35	0.84	1271	1013	1.25
	10mg[f] [57]	3.70	4.35	0.85	1300	1013	1.28
captopril	10mg[a] [60]	0.102	0.141	0.72	69.20	79.10	0.87
	25mg[a] [61]	0.337	0.352	0.96	235.20	197.70	1.19
	25mg[g] [62]	0.364	0.352	1.03	162.90	197.70	0.82
	100mg[a][63]	1.15	1.41	0.82	800	790.60	1.01
	100mg[g] [64]	1.32	1.33	0.99	930	790.60	1.18
	100mg[f] [65]	1.39	1.38	1.01	803	790.60	1.02
valsartan	80mg[a] [67]	16.10	26.30	0.61	3000	2644.78	1.13
	160mg[b] [68]	33.18	52.63	0.63	6110	5289.56	1.16
	160mg[b] [68]	33.87	52.63	0.64	5940	5289.56	1.12
	160mg[b] [68]	34.52	52.63	0.66	5940	5289.56	1.12
felodipine	5mg[a] [70]	0.013	0.019	0.68	2.38	2.54	0.94
	5mg[h] [71]	0.0085	0.012	0.71	3.07	2.54	1.21
	10mg[f] [33]	0.029	0.036	0.81	6.95	5.08	1.37
	10mg[f] [72]	0.025	0.030	0.83	5.57	5.08	1.10
midazolam	3mg[a] [74]	0.035	0.045	0.78	15	17.47	0.86
	7.5mg[a] [35]	0.10	0.13	0.77	42	46.96	0.89
	7.5mg[a] [75]	0.10	0.13	0.77	34	46.96	0.72
	7.5mg[a] [76]	0.043	0.13	0.33	41.50	46.96	0.88

Specific time of $AUC_{0\text{-}t}$: [a]: $AUC_{0\text{-}\infty}$; [b]: 24h; [c]: 10h; [d]: 48h; [e]: 120h; [f]: 12h; [g]: 6h; [h]: 8h.

References

1. Edginton, A. N.; Willmann, S. Physiology-based simulations of a pathological condition: prediction of pharmacokinetics in patients with liver cirrhosis. *Clin Pharmacokinet* **2008**, *47* (11), 743-752. https://doi.org/10.2165/00003088-200847110-00005
2. Yu, L. X.; Amidon, G. L. A compartmental absorption and transit model for estimating oral drug absorption. *Int J Pharm* **1999**, *186* (2), 119-125. https://doi.org/10.1016/s0378-5173(99)00147-7
3. Gertz, M.; Houston, J. B.; Galetin, A. Physiologically based pharmacokinetic modeling of intestinal first-pass metabolism of CYP3A substrates with high intestinal extraction. *Drug Metab Dispos* **2011**, *39* (9), 1633-1642. https://doi.org/10.1124/dmd.111.039248
4. Scalese, M. J.; Salvatore, D. J. Role of Digoxin in Atrial Fibrillation. *J Pharm Pract* **2017**, *30* (4), 434-440. https://doi.org/10.1177/0897190016642361
5. Lainscak, M.; Vitale, C.; Seferovic, P.; Spoletini, I.; Cvan Trobec, K.; Rosano, G. M. Pharmacokinetics and pharmacodynamics of cardiovascular drugs in chronic heart failure. *Int J Cardiol* **2016**, *224*, 191-198. https://doi.org/10.1016/j.ijcard.2016.09.015
6. Neuhoff, S.; Yeo, K. R.; Barter, Z.; Jamei, M.; Turner, D. B.; Rostami-Hodjegan, A. Application of permeability-limited physiologically-based pharmacokinetic models: part I-digoxin pharmacokinetics incorporating P-glycoprotein-mediated efflux. *J Pharm Sci* **2013**, *102* (9), 3145-3160. https://doi.org/10.1002/jps.23594
7. Solanki, D.; Choudhary, S.; Vora, A.; Ghose, T.; Mantri, R. R.; Modi, N.; Sawhney, J.; Singhal, A.; Kumar, A.; Edakutty, R.; et al. Loop Diuretics Unique Mechanism of Action. *J Assoc Physicians India* **2024**, *72* (9s), 14-15. https://doi.org/10.59556/japi.72.0670
8. Ponto, L. L.; Schoenwald, R. D. Furosemide (frusemide). A pharmacokinetic/pharmacodynamic review (Part I). *Clin Pharmacokinet* **1990**, *18* (5), 381-408. https://doi.org/10.2165/00003088-199018050-00004

9. Chapa, R.; Li, C. Y.; Basit, A.; Thakur, A.; Ladumor, M. K.; Sharma, S.; Singh, S.; Selen, A.; Prasad, B. Contribution of Uptake and Efflux Transporters to Oral Pharmacokinetics of Furosemide. *ACS Omega* **2020**, *5* (51), 32939-32950. https://doi.org/10.1021/acsomega.0c03930
10. Donovan, M. D.; Abduljalil, K.; Cryan, J. F.; Boylan, G. B.; Griffin, B. T. Application of a physiologically-based pharmacokinetic model for the prediction of bumetanide plasma and brain concentrations in the neonate. *Biopharm Drug Dispos* **2018**, *39* (3), 125-134. https://doi.org/10.1002/bdd.2119
11. Kramer, W. G. Pharmacokinetics and pharmacodynamics of torasemide in congestive heart failure. *Cardiology* **1994**, *84 Suppl 2*, 108-114. https://doi.org/10.1159/000176463
12. Knauf, H.; Mutschler, E. Clinical pharmacokinetics and pharmacodynamics of torasemide. *Clin Pharmacokinet* **1998**, *34* (1), 1-24. https://doi.org/10.2165/00003088-199834010-00001
13. Brogden, R. N.; Todd, P. A.; Sorkin, E. M. Captopril. An update of its pharmacodynamic and pharmacokinetic properties, and therapeutic use in hypertension and congestive heart failure. *Drugs* **1988**, *36* (5), 540-600. https://doi.org/10.2165/00003495-198836050-00003
14. Rasool, M. F.; Ali, S.; Khalid, S.; Khalid, R.; Majeed, A.; Imran, I.; Saeed, H.; Usman, M.; Ali, M.; Alali, A. S.; et al. Development and evaluation of physiologically based pharmacokinetic drug-disease models for predicting captopril pharmacokinetics in chronic diseases. *Sci Rep* **2021**, *11* (1), 8589. https://doi.org/10.1038/s41598-021-88154-2
15. Michel, M. C.; Foster, C.; Brunner, H. R.; Liu, L. A systematic comparison of the properties of clinically used angiotensin II type 1 receptor antagonists. *Pharmacol Rev* **2013**, *65* (2), 809-848. https://doi.org/10.1124/pr.112.007278
16. Zhuang, X.; Lu, C. PBPK modeling and simulation in drug research and development. *Acta Pharm Sin B* **2016**, *6* (5), 430-440. https://doi.org/10.1016/j.apsb.2016.04.004
17. Yedinak, K. C.; Lopez, L. M. Felodipine: a new dihydropyridine calcium-channel antagonist. *Dicp* **1991**, *25* (11), 1193-1206. https://doi.org/10.1177/106002809102501109
18. Salem, F.; Nimavardi, A.; Mudunuru, J.; Tompson, D.; Bloomer, J.; Turner, D. B.; Taskar, K. S. Physiologically based pharmacokinetic modeling for development and applications of a virtual celiac disease population using felodipine as a model drug. *CPT Pharmacometrics Syst Pharmacol* **2023**, *12* (6), 808-820. https://doi.org/10.1002/psp4.12954
19. Kong, W. M.; Sun, B. B.; Wang, Z. J.; Zheng, X. K.; Zhao, K. J.; Chen, Y.; Zhang, J. X.; Liu, P. H.; Zhu, L.; Xu, R. J.; et al. Physiologically based pharmacokinetic-pharmacodynamic modeling for prediction of vonoprazan pharmacokinetics and its inhibition on gastric acid secretion following intravenous/oral administration to rats, dogs and humans. *Acta Pharmacol Sin* **2020**, *41* (6), 852-865. https://doi.org/10.1038/s41401-019-0353-2
20. Yang, Y.; Li, P.; Zhang, Z.; Wang, Z.; Liu, L.; Liu, X. Prediction of Cyclosporin-Mediated Drug Interaction Using Physiologically Based Pharmacokinetic Model Characterizing Interplay of Drug Transporters and Enzymes. *Int J Mol Sci* **2020**, *21* (19). https://doi.org/10.3390/ijms21197023
21. Jeong, S. H.; Jang, J. H.; Lee, Y. B. Torsemide Pharmacometrics in Healthy Adult Populations Including CYP2C9 Genetic Polymorphisms and Various Patient Groups through Physiologically Based Pharmacokinetic-Pharmacodynamic Modeling. *Pharmaceutics* **2022**, *14* (12). https://doi.org/10.3390/pharmaceutics14122720
22. McPherson, S.; Perrier, J.; Dunn, C.; Khadra, I.; Davidson, S.; Ainousah, B.; Wilson, C. G.; Halbert, G. Small scale design of experiment investigation of equilibrium solubility in simulated fasted and fed intestinal fluid. *Eur J Pharm Biopharm* **2020**, *150*, 14-23. https://doi.org/10.1016/j.ejpb.2020.01.016
23. Fuhr, L. M.; Marok, F. Z.; Mees, M.; Mahfoud, F.; Selzer, D.; Lehr, T. A Physiologically Based Pharmacokinetic and Pharmacodynamic Model of the CYP3A4 Substrate Felodipine for Drug-Drug Interaction Modeling. *Pharmaceutics* **2022**, *14* (7). https://doi.org/10.3390/pharmaceutics14071474
24. Moj, D.; Hanke, N.; Britz, H.; Frechen, S.; Kanacher, T.; Wendl, T.; Haefeli, W. E.; Lehr, T. Clarithromycin, Midazolam, and Digoxin: Application of PBPK Modeling to Gain New Insights into Drug-Drug Interactions and Co-medication Regimens. *Aaps j* **2017**, *19* (1), 298-312. https://doi.org/10.1208/s12248-016-0009-9
25. Schlender, J. F.; Meyer, M.; Thelen, K.; Krauss, M.; Willmann, S.; Eissing, T.; Jaehde, U. Development of a Whole-Body Physiologically Based Pharmacokinetic Approach to Assess the Pharmacokinetics of Drugs in Elderly Individuals. *Clin Pharmacokinet* **2016**, *55* (12), 1573-1589. https://doi.org/10.1007/s40262-016-0422-3
26. Poirier, A.; Cascais, A. C.; Funk, C.; Lavé, T. Prediction of pharmacokinetic profile of valsartan in human based on in vitro uptake transport data. *J Pharmacokinet Pharmacodyn* **2009**, *36* (6), 585-611. https://doi.org/10.1007/s10928-009-9139-3

27. Pandey, M. M.; Jaipal, A.; Kumar, A.; Malik, R.; Charde, S. Y. Determination of pK(a) of felodipine using UV-Visible spectroscopy. *Spectrochim Acta A Mol Biomol Spectrosc* **2013**, *115*, 887-890. https://doi.org/10.1016/j.saa.2013.07.001
28. Hanke, N.; Frechen, S.; Moj, D.; Britz, H.; Eissing, T.; Wendl, T.; Lehr, T. PBPK Models for CYP3A4 and P-gp DDI Prediction: A Modeling Network of Rifampicin, Itraconazole, Clarithromycin, Midazolam, Alfentanil, and Digoxin. *CPT Pharmacometrics Syst Pharmacol* **2018**, *7* (10), 647-659. https://doi.org/10.1002/psp4.12343
29. Tsutsumi, K.; Kotegawa, T.; Kuranari, M.; Otani, Y.; Morimoto, T.; Matsuki, S.; Nakano, S. The effect of erythromycin and clarithromycin on the pharmacokinetics of intravenous digoxin in healthy volunteers. *J Clin Pharmacol* **2002**, *42* (10), 1159-1164. https://doi.org/10.1177/009127002401382641
30. Cook, J. A.; Smith, D. E.; Cornish, L. A.; Tankanow, R. M.; Nicklas, J. M.; Hyneck, M. L. Kinetics, dynamics, and bioavailability of bumetanide in healthy subjects and patients with congestive heart failure. *Clin Pharmacol Ther* **1988**, *44* (5), 487-500. https://doi.org/10.1038/clpt.1988.186
31. Schwartz, S.; Brater, D. C.; Pound, D.; Green, P. K.; Kramer, W. G.; Rudy, D. Bioavailability, pharmacokinetics, and pharmacodynamics of torsemide in patients with cirrhosis. *Clin Pharmacol Ther* **1993**, *54* (1), 90-97. https://doi.org/10.1038/clpt.1993.116
32. Flesch, G.; Müller, P.; Lloyd, P. Absolute bioavailability and pharmacokinetics of valsartan, an angiotensin II receptor antagonist, in man. *Eur J Clin Pharmacol* **1997**, *52* (2), 115-120. https://doi.org/10.1007/s002280050259
33. Larsson, R.; Karlberg, B. E.; Gelin, A.; Aberg, J.; Regårdh, C. G. Acute and steady-state pharmacokinetics and antihypertensive effects of felodipine in patients with normal and impaired renal function. *J Clin Pharmacol* **1990**, *30* (11), 1020-1030. https://doi.org/10.1002/j.1552-4604.1990.tb03589.x
34. Rowland Yeo, K.; Walsky, R. L.; Jamei, M.; Rostami-Hodjegan, A.; Tucker, G. T. Prediction of time-dependent CYP3A4 drug-drug interactions by physiologically based pharmacokinetic modelling: impact of inactivation parameters and enzyme turnover. *Eur J Pharm Sci* **2011**, *43* (3), 160-173. https://doi.org/10.1016/j.ejps.2011.04.008
35. Patel, I. H.; Soni, P. P.; Fukuda, E. K.; Smith, D. F.; Leier, C. V.; Boudoulas, H. The pharmacokinetics of midazolam in patients with congestive heart failure. *Br J Clin Pharmacol* **1990**, *29* (5), 565-569. https://doi.org/10.1111/j.1365-2125.1990.tb03680.x
36. Qian, C. Q.; Zhao, K. J.; Chen, Y.; Liu, L.; Liu, X. D. Simultaneously predict pharmacokinetic interaction of rifampicin with oral versus intravenous substrates of cytochrome P450 3A/P-glycoprotein to healthy human using a semi-physiologically based pharmacokinetic model involving both enzyme and transporter turnover. *Eur J Pharm Sci* **2019**, *134*, 194-204. https://doi.org/10.1016/j.ejps.2019.04.026
37. Rodgers, T.; Rowland, M. Physiologically based pharmacokinetic modelling 2: predicting the tissue distribution of acids, very weak bases, neutrals and zwitterions. *J Pharm Sci* **2006**, *95* (6), 1238-1257. https://doi.org/10.1002/jps.20502
38. Ruark, C. D.; Hack, C. E.; Robinson, P. J.; Mahle, D. A.; Gearhart, J. M. Predicting passive and active tissue:plasma partition coefficients: interindividual and interspecies variability. *J Pharm Sci* **2014**, *103* (7), 2189-2198. https://doi.org/10.1002/jps.24011
39. Eckermann, G.; Lahu, G.; Nassr, N.; Bethke, T. D. Absence of pharmacokinetic interaction between roflumilast and digoxin in healthy adults. *J Clin Pharmacol* **2012**, *52* (2), 251-257. https://doi.org/10.1177/0091270010389467
40. Gurley, B. J.; Swain, A.; Williams, D. K.; Barone, G.; Battu, S. K. Gauging the clinical significance of P-glycoprotein-mediated herb-drug interactions: comparative effects of St. John's wort, Echinacea, clarithromycin, and rifampin on digoxin pharmacokinetics. *Mol Nutr Food Res* **2008**, *52* (7), 772-779. https://doi.org/10.1002/mnfr.200700081
41. Martin, P. D.; Kemp, J.; Dane, A. L.; Warwick, M. J.; Schneck, D. W. No effect of rosuvastatin on the pharmacokinetics of digoxin in healthy volunteers. *J Clin Pharmacol* **2002**, *42* (12), 1352-1357. https://doi.org/10.1177/0091270002042012008
42. Verstuyft, C.; Strabach, S.; El-Morabet, H.; Kerb, R.; Brinkmann, U.; Dubert, L.; Jaillon, P.; Funck-Brentano, C.; Trugnan, G.; Becquemont, L. Dipyridamole enhances digoxin bioavailability via P-glycoprotein inhibition. *Clin Pharmacol Ther* **2003**, *73* (1), 51-60. https://doi.org/10.1067/mcp.2003.8
43. Tarral, A.; Francheteau, P.; Guerret, M. Effects of terbinafine on the pharmacokinetics of digoxin in healthy volunteers. *Pharmacotherapy* **1997**, *17* (4), 791-795.
44. Zhou, H.; Horowitz, A.; Ledford, P. C.; Hubert, M.; Appel-Dingemanse, S.; Osborne, S.; McLeod, J. F. The effects of tegaserod (HTF 919) on the pharmacokinetics and pharmacodynamics of digoxin in healthy subjects. *J Clin Pharmacol* **2001**, *41* (10), 1131-1139. https://doi.org/10.1177/00912700122012625

45. Hayward, R. P.; Greenwood, H.; Hamer, J. Comparison of digoxin and medigoxin in normal subjects. *Br J Clin Pharmacol* **1978**, *6* (1), 81-86. https://doi.org/10.1111/j.1365-2125.1978.tb01686.x
46. Ohnhaus, E. E.; Vozeh, S.; Nuesch, E. Absorption of digoxin in severe right heart failure. *Eur J Clin Pharmacol* **1979**, *15* (2), 115-120. https://doi.org/10.1007/bf00609874
47. Liu, Z.; Fang, S.; Wang, L.; Zhu, T.; Yang, H.; Yu, S. Clinical study on chronopharmacokinetics of digoxin in patients with congestive heart failure. *J Tongji Med Univ* **1998**, *18* (1), 21-24. https://doi.org/10.1007/bf02888273
48. Britz, H.; Hanke, N.; Taub, M. E.; Wang, T.; Prasad, B.; Fernandez, É.; Stopfer, P.; Nock, V.; Lehr, T. Physiologically Based Pharmacokinetic Models of Probenecid and Furosemide to Predict Transporter Mediated Drug-Drug Interactions. *Pharm Res* **2020**, *37* (12), 250. https://doi.org/10.1007/s11095-020-02964-z
49. Andreasen, F.; Christensen, C. K.; Jacobsen, F. K.; Jansen, J.; Mogensen, C. E.; Pedersen, O. L. The individual variation in pharmacokinetics and pharmacodynamics of furosemide in young normal male subjects. *Eur J Clin Invest* **1982**, *12* (3), 247-255. https://doi.org/10.1111/j.1365-2362.1982.tb01000.x
50. Verbeeck, R. K.; Patwardhan, R. V.; Villeneuve, J. P.; Wilkinson, G. R.; Branch, R. A. Furosemide disposition in cirrhosis. *Clin Pharmacol Ther* **1982**, *31* (6), 719-725. https://doi.org/10.1038/clpt.1982.101
51. Kelly, M. R.; Cutler, R. E.; Forrey, A. W.; Kimpel, B. M. Pharmacokinetics of orally administered furosemide. *Clin Pharmacol Ther* **1974**, *15* (2), 178-186.
52. Smith, D. E.; Gee, W. L.; Brater, D. C.; Lin, E. T.; Benet, L. Z. Preliminary evaluation of furosemide-probenecid interaction in humans. *J Pharm Sci* **1980**, *69* (5), 571-575. https://doi.org/10.1002/jps.2600690526
53. Keller, E.; Hoppe-Seyler, G.; Mumm, R.; Schollmeyer, P. Influence of hepatic cirrhosis and end-stage renal disease on pharmacokinetics and pharmacodynamics of furosemide. *Eur J Clin Pharmacol* **1981**, *20* (1), 27-33. https://doi.org/10.1007/bf00554663
54. Hammarlund, M. M.; Paalzow, L. K.; Odlind, B. Pharmacokinetics of furosemide in man after intravenous and oral administration. Application of moment analysis. *Eur J Clin Pharmacol* **1984**, *26* (2), 197-207. https://doi.org/10.1007/bf00630286
55. Andreasen, F.; Mikkelsen, E. Distribution, elimination and effect of furosemide in normal subjects and in patients with heart failure. *Eur J Clin Pharmacol* **1977**, *12* (1), 15-22. https://doi.org/10.1007/bf00561400
56. Müller, F. O.; Middle, M. V.; Schall, R.; Terblanché, J.; Hundt, H. K.; Groenewoud, G. An evaluation of the interaction of meloxicam with frusemide in patients with compensated chronic cardiac failure. *Br J Clin Pharmacol* **1997**, *44* (4), 393-398. https://doi.org/10.1046/j.1365-2125.1997.t01-1-00586.x
57. Vargo, D. L.; Kramer, W. G.; Black, P. K.; Smith, W. B.; Serpas, T.; Brater, D. C. Bioavailability, pharmacokinetics, and pharmacodynamics of torsemide and furosemide in patients with congestive heart failure. *Clin Pharmacol Ther* **1995**, *57* (6), 601-609. https://doi.org/10.1016/0009-9236(95)90222-8
58. Holazo, A. A.; Colburn, W. A.; Gustafson, J. H.; Young, R. L.; Parsonnet, M. Pharmacokinetics of bumetanide following intravenous, intramuscular, and oral administrations to normal subjects. *J Pharm Sci* **1984**, *73* (8), 1108-1113. https://doi.org/10.1002/jps.2600730821
59. Lau, H. S.; Hyneck, M. L.; Berardi, R. R.; Swartz, R. D.; Smith, D. E. Kinetics, dynamics, and bioavailability of bumetanide in healthy subjects and patients with chronic renal failure. *Clin Pharmacol Ther* **1986**, *39* (6), 635-645. https://doi.org/10.1038/clpt.1986.112
60. Duchin, K. L.; Singhvi, S. M.; Willard, D. A.; Migdalof, B. H.; McKinstry, D. N. Captopril kinetics. *Clin Pharmacol Ther* **1982**, *31* (4), 452-458. https://doi.org/10.1038/clpt.1982.59
61. Chik, Z.; Basu, R. C.; Pendek, R.; Lee, T. C.; Mohamed, Z. A bioequivalence comparison of two formulations of rifampicin (300- vs 150-mg capsules): An open-label, randomized, two-treatment, two-way crossover study in healthy volunteers. *Clin Ther* **2010**, *32* (10), 1822-1831. https://doi.org/10.1016/j.clinthera.2010.09.006
62. Jankowski, A.; Skorek, A.; Krzyśko, K.; Zarzycki, P. K.; Ochocka, R. J.; Lamparczyk, H. Captopril: determination in blood and pharmacokinetics after single oral dose. *J Pharm Biomed Anal* **1995**, *13* (4-5), 655-660. https://doi.org/10.1016/0731-7085(95)01319-g
63. Kripalani, K. J.; McKinstry, D. N.; Singhvi, S. M.; Willard, D. A.; Vukovich, R. A.; Migdalof, B. H. Disposition of captopril in normal subjects. *Clin Pharmacol Ther* **1980**, *27* (5), 636-641. https://doi.org/10.1038/clpt.1980.90
64. Singhvi, S. M.; McKinstry, D. N.; Shaw, J. M.; Willard, D. A.; Migdalof, B. H. Effect of food on the bioavailability of captopril in healthy subjects. *J Clin Pharmacol* **1982**, *22* (2-3), 135-140. https://doi.org/10.1002/j.1552-4604.1982.tb02661.x

65. Creasey, W. A.; Funke, P. T.; McKinstry, D. N.; Sugerman, A. A. Pharmacokinetics of captopril in elderly healthy male volunteers. *J Clin Pharmacol* **1986**, *26* (4), 264-268. https://doi.org/10.1002/j.1552-4604.1986.tb03521.x
66. Shaw, T. R.; Duncan, F. M.; Williams, B. C.; Crichton, E.; Thomson, S. A.; Davis, J. R.; Rademaker, M.; Edwards, C. R. Plasma free captopril concentrations during short and long term treatment with oral captopril for heart failure. *Br Heart J* **1985**, *54* (2), 160-165. https://doi.org/10.1136/hrt.54.2.160
67. Sioufi, A.; Marfil, F.; Jaouen, A.; Cardot, J. M.; Godbillon, J.; Ezzet, F.; Lloyd, P. The effect of age on the pharmacokinetics of valsartan. *Biopharm Drug Dispos* **1998**, *19* (4), 237-244. https://doi.org/10.1002/(sici)1099-081x(199805)19:4<237::aid-bdd100>3.0.co;2-7
68. Iqbal, M.; Khuroo, A.; Batolar, L. S.; Tandon, M.; Monif, T.; Sharma, P. L. Pharmacokinetics and bioequivalence study of three oral formulations of valsartan 160 mg: a single-dose, randomized, open-label, three-period crossover comparison in healthy Indian male volunteers. *Clin Ther* **2010**, *32* (3), 588-596. https://doi.org/10.1016/j.clinthera.2010.03.004
69. Prasad, P. P.; Yeh, C. M.; Gurrieri, P.; Glazer, R.; McLeod, J. Pharmacokinetics of multiple doses of valsartan in patients with heart failure. *J Cardiovasc Pharmacol* **2002**, *40* (5), 801-807. https://doi.org/10.1097/00005344-200211000-00018
70. Landahl, S.; Edgar, B.; Gabrielsson, M.; Larsson, M.; Lernfelt, B.; Lundborg, P.; Regårdh, C. G. Pharmacokinetics and blood pressure effects of felodipine in elderly hypertensive patients. A comparison with young healthy subjects. *Clin Pharmacokinet* **1988**, *14* (6), 374-383. https://doi.org/10.2165/00003088-198814060-00004
71. Bailey, D. G.; Arnold, J. M.; Munoz, C.; Spence, J. D. Grapefruit juice--felodipine interaction: mechanism, predictability, and effect of naringin. *Clin Pharmacol Ther* **1993**, *53* (6), 637-642. https://doi.org/10.1038/clpt.1993.84
72. Hardy, B. G.; Bartle, W. R.; Myers, M.; Bailey, D. G.; Edgar, B. Effect of indomethacin on the pharmacokinetics and pharmacodynamics of felodipine. *Br J Clin Pharmacol* **1988**, *26* (5), 557-562. https://doi.org/10.1111/j.1365-2125.1988.tb05295.x
73. Rehnqvist, N.; Billing, E.; Moberg, L.; Lundman, T.; Olsson, G. Pharmacokinetics of felodipine and effect on digoxin plasma levels in patients with heart failure. *Drugs* **1987**, *34 Suppl 3*, 33-42. https://doi.org/10.2165/00003495-198700343-00009
74. Kharasch, E. D.; Francis, A.; London, A.; Frey, K.; Kim, T.; Blood, J. Sensitivity of intravenous and oral alfentanil and pupillary miosis as minimal and noninvasive probes for hepatic and first-pass CYP3A induction. *Clin Pharmacol Ther* **2011**, *90* (1), 100-108. https://doi.org/10.1038/clpt.2011.59
75. Ahonen, J.; Olkkola, K. T.; Neuvonen, P. J. Effect of itraconazole and terbinafine on the pharmacokinetics and pharmacodynamics of midazolam in healthy volunteers. *Br J Clin Pharmacol* **1995**, *40* (3), 270-272.
76. Abdlekawy, K. S.; Donia, A. M.; Elbarbry, F. Effects of Grapefruit and Pomegranate Juices on the Pharmacokinetic Properties of Dapoxetine and Midazolam in Healthy Subjects. *Eur J Drug Metab Pharmacokinet* **2017**, *42* (3), 397-405. https://doi.org/10.1007/s13318-016-0352-3
